# Radezolid Is More Effective Than Linezolid Against Planktonic Cells and Inhibits *Enterococcus faecalis* Biofilm Formation

**DOI:** 10.3389/fmicb.2020.00196

**Published:** 2020-02-14

**Authors:** Jinxin Zheng, Zhong Chen, Zhiwei Lin, Xiang Sun, Bing Bai, Guangjian Xu, Junwen Chen, Zhijian Yu, Di Qu

**Affiliations:** ^1^Key Laboratory of Medical Molecular Virology of Ministries of Education and Health, School of Basic Medical Science and Institutes of Biomedical Sciences, Shanghai Medical College of Fudan University, Shanghai, China; ^2^Department of Infectious Diseases and the Key Laboratory of Endogenous Infection, Shenzhen Nanshan People’s Hospital, The 6th Affiliated Hospital of Shenzhen University Health Science Center, Shenzhen, China

**Keywords:** *Enterococcus faecalis*, radezolid, linezolid, resistance, biofilm, efflux pump

## Abstract

The aim of this study was to compare the effects of radezolid and linezolid on planktonic and biofilm cells of *Enterococcus faecalis*. A total of 302 *E. faecalis* clinical isolates were collected, and the minimum inhibitory concentrations (MICs) of radezolid and linezolid were determined by the agar dilution method. Changes in the transcriptome of a high-level, *in vitro*-induced linezolid-resistant isolate were assessed by RNA sequencing and RT-qPCR, and the roles of efflux pump-related genes were confirmed by overexpression analysis. Biofilm biomass was evaluated by crystal violet staining and the adherent cells in the biofilms were quantified according to CFU numbers. The MIC_50_/MIC_90_ values of radezolid (0.25/0.50 mg/L) against the 302 *E. faecalis* clinical isolates were eightfold lower than those of linezolid (2/4 mg/L). The radezolid MICs against the high-level linezolid-resistant isolates (linezolid MICs ≥ 64 mg/L) increased to ≥ 4 mg/L with mutations in the four copies of the V domain of the 23S rRNA gene. The mRNA expression level of *OG1RF_12220* (*mdlB2*, multidrug ABC superfamily ATP-binding cassette transporter) increased in the high-level linezolid-resistant isolates, and radezolid and linezolid MICs against the linezolid-sensitive isolate increased with overexpression of *OG1RF_12220*. Radezolid (at 1/4 or 1/8× the MIC) inhibited *E. faecalis* biofilm formation to a greater extent than linezolid, which was primarily achieved through the inhibition of *ahrC*, *esp*, *relA*, and *relQ* transcription in *E. faecalis*. In conclusion, radezolid is more effective than linezolid against planktonic *E. faecalis* cells and inhibits biofilm formation by this bacterium.

## Introduction

*Enterococcus faecalis* is a prominent example of a human pathogen that rapidly evolves and becomes refractory to a wide range of antimicrobials. In addition to the intrinsic and acquired resistance to many individual antimicrobials, the spread of multidrug-resistant (MDR) enterococci, especially those resistant to vancomycin (VRE), has further narrowed the choices for anti-infective therapy (Ahmed and Baptiste, 2018). Linezolid (LZD), an important member of the oxazolidinone class of antibiotics, has proven to be highly effective against most gram-positive bacteria and is recommended as the first-line choice for the remedial treatment of VRE and other MDR enterococci infections (Whang et al., 2013). However, widespread LZD application has led to the rapid, global emergence of LZD-resistant clinical isolates, including *Staphylococcus aureus*, *Staphylococcus epidermidis*, *E. faecalis, E. faecium, Mycobacterium tuberculosis*, and *Mycobacterium abscessus* (Balandin et al., 2016; Zimenkov et al., 2017; Chen et al., 2018; Silva et al., 2019; Ye et al., 2019). The consequent renewed interest in the optimization of oxazolidinones led to the development of new antimicrobials such as radezolid (RZD, RX-1741) (Lemaire et al., 2010b), which showed greater potency than LZD against a broad range of gram-positive bacteria, including VRE (Lemaire et al., 2010a; Wu et al., 2018, 2019). However, whether RZD is also effective against linezolid-resistant *E. faecalis* isolates remains unclear.

Numerous studies have demonstrated that LZD resistance is associated with mutations in domain V of the 23S rRNA gene and L3 and L4 ribosomal proteins, as well as with the acquisition of the *cfr*, *cfr(B)*, or *optrA* genes (Sadowy, 2018). Recently, the ABC-F subfamily ATP-binding cassette protein PoxtA was also found to play a role in the decreased susceptibility of *S. aureus* and *E. faecalis* to oxazolidinones (Antonelli et al., 2018; Elghaieb et al., 2019; Hasman et al., 2019; Lei et al., 2019). Nevertheless, the extent to which RZD exerts enhanced antibacterial activity against *E. faecalis* when compared with LZD is still not known. Additionally, LZD has been reported to have good inhibitory effects on *E. faecalis* biofilms (Holmberg et al., 2012); however, it is also unclear whether RZD shows greater efficacy than LZD against *E. faecalis* biofilms. To address these questions, in this study, we compared the antibacterial effects of RZD and LZD against biofilm and planktonic cells of *E. faecalis*.

## Materials and Methods

### Bacterial Strains and Antimicrobials

A total of 302 non-duplicate *E. faecalis* isolates were collected from different inpatients at Shenzhen Nanshan People’s Hospital (Grade A, level III Hospital, 1500 beds), Shenzhen University, China, between January 1, 2011, and December 31, 2016. These *E. faecalis* isolates were obtained from urine (135 isolates), blood (37 isolates), pus or secretions (86 isolates), bile (25 isolates), and other clinical sources (19 isolates). Based on a previous study, the dominant multilocus sequence types (MLSTs) of these isolates were ST16 and ST179 (Zheng et al., 2017). The isolates were identified by the Phoenix 100 automated microbiology system (BD, Franklin Lakes, NJ, United States), following which two subcultured generations of all the 302 isolates were re-identified with matrix-assisted laser desorption ionization time-of-flight mass spectrometry (IVD MALDI Biotyper, Bruker, Bremen, Germany). *E. faecalis* strains ATCC29212 and OG1RF (ATCC47077) were used as reference strains.

Chloramphenicol (catalog no. HY-B0239), linezolid (catalog no. HY-10394), and radezolid (catalog no. HY-14800) were purchased from MedChemExpress (MCE, Shanghai, China).

### Antimicrobial Susceptibility Test and Detection of LZD Resistance Genes

The susceptibilities of *E. faecalis* isolates to clinically relevant antimicrobials were tested by the Phoenix 100 automated microbiology system. The minimum inhibitory concentrations (MICs) of LZD and RZD were determined by the agar dilution method according to Clinical and Laboratory Standards Institute (CLSI) guidelines. While no CLSI interpretive criteria existed for RZD against enterococci, the MICs of RZD for the quality control strain ATCC29212 were observed to range from 0.06 to 0.5 mg/L. Therefore, to analyze the distribution of MICs for RZD in these clinical isolates, the MICs were categorized into the following four levels: ≤ 0.125, 0.25, 0.5, and ≥ 1 mg/L. The four copies of the V domain of the 23S rRNA gene, as well as the *rplC* and *rplD* genes, were amplified by PCR and sequenced. The *cfr*, *cfr(B)*, *optrA*, and *poxtA* genes were also amplified by PCR. The primers used for PCR are listed in Supplementary Table S1.

### *In vitro* Induction of High-Level LZD-Resistant Isolates and Efflux Inhibition Assay

The ATCC29212-0 and OG1RF (ATCC47077)-0 strains (LZD-sensitive, linezolid MIC: 2 mg/L) were serially subcultured in Mueller–Hinton Broth (MHB) containing LZD. The initial inducing concentration of LZD was 0.5× the MIC, which was then successively increased to 1, 2, 4, 8, 16, 32, 64, and 128× the MIC. Strains were cultured at each concentration for 3–5 passages before their exposure to the next concentration. Isolates from the final passage of each concentration were identified by matrix-assisted laser desorption ionization time-of-flight mass spectrometry (IVD MALDI Biotyper, Bruker, Bremen, Germany), and the MICs of RZD and LZD were determined by the agar dilution method according to CLSI guidelines.

The efflux pump activities in LZD-resistant isolates were detected using the efflux pump inhibitor Phe-Arg-β-naphthylamide (PAβN, Sigma, Shanghai, China). MICs for RZD and LZD were determined in the presence or absence of PAβN (20 mg/L) (Kothary et al., 2013). This assay was performed at least in triplicate.

### RNA Isolation and Sequencing

Total RNA isolation and RNA sequencing (RNA-seq) of the *E. faecalis* OG1RF wild-type isolate (OG1RF-0, LZD- and RZD-sensitive) and the high-level LZD-resistant isolate OG1RF-55 (linezolid MIC: 256 mg/L; radezolid MIC: 8 mg/L) were performed as previously described (Wang et al., 2017). Briefly, planktonic *E. faecalis* cells were homogenized using 0.1-mm zirconia–silica beads in a mini-BeadBeater and the total RNA in the supernatant was purified using an RNeasy Mini Kit (Qiagen, Hilden, Germany). RNA-seq was performed according to the Illumina RNA sequencing sample preparation guide. Total RNA samples were treated with RNase-free DNase I (TaKaRa Biotechnology, Dalian, China). cDNA libraries were prepared using an RNA-seq sample preparation kit (Illumina, San Diego, CA, United States), and sequencing was performed with an Illumina HiSeq 2500 sequencer for 50 cycles. All these procedures were performed according to the manufacturers’ protocols. Raw sequencing data were processed using the data collection software provided by Illumina. RNA-seq was performed in three independent experiments.

### RNA-Seq Data Analysis and RT-qPCR

Raw sequencing reads were preprocessed by filtering out rRNA reads, sequencing adapters, short fragment reads, and other low-quality reads. The remaining reads were mapped to the *E. faecalis* OG1RF reference genome (CP002621.1) at the National Center for Biotechnology Information (NCBI) website using Bowtie2 software (version 2.0.5) based on the local alignment algorithm. The alignments reported using Bowtie2 software were further processed with BED Tools software to determine transcript expression levels and their differential expression between each two of the three samples. Differential expression of all the transcripts was quantified using DEGseq software (version 2.16.1), and then the fold-change values were presented. For validation of the RNA-seq results, RT-qPCR was performed using the SYBR Premix Ex Taq II Kit (TaKaRa Biotechnology, Dalian, China) on a Mastercycler ep realplex system (Eppendorf, Hamburg, Germany) as previously described (Zheng et al., 2017). The primers used for RT-qPCR are listed in Supplementary Table S2. RT-qPCR was performed in triplicate at least three times.

### Overexpression of Efflux Pump-Related Genes in *E. faecalis*

The *OG1RF_12220* gene (multidrug ABC superfamily ATP-binding cassette transporter, *mdlB2*) and three ABC superfamily ATP-binding cassette transporter genes (*OG1RF_10126, OG1RF_10665*, and *OG1RF_10495*) were amplified by PCR. The amplicons were purified and digested with endonucleases, and then cloned into the pIB166 plasmid for gene overexpression. Correct cloning was verified by PCR and sequencing. Verified loaded plasmids were introduced into the *E. faecalis* OG1RF strain. All strains, plasmids, and primers used for overexpression analysis are listed in Supplementary Tables S3, S4.

### Biofilm Biomass Assay and Adherent Cell Detection

The biofilm biomass of 13 *E. faecalis* clinical isolates (16C1, 16C35, 16C51, 16C102, 16C106, 16C124, 16C138, 16C152, 16C166, 16C201, 16C289, 16C350, and 16C353) was detected by crystal violet staining as previously described (Zheng et al., 2019). For analysis of the eradication potential of RZD or LZD against established *E. faecalis* biofilms, *E. faecalis* isolates were inoculated into 96-well polystyrene microtiter plates with TSBG (tryptic soy broth with 0.25% glucose) for formation of mature biofilms. After 24 h of static incubation, the supernatants were discarded and plates were washed with 0.9% saline to remove unattached cells, following which fresh TSBG containing RZD or LZD was added. After 48 h of static incubation, with the medium replaced daily, the remaining biofilm biomass was determined by crystal violet staining. The numbers of adherent cells remaining in biofilms formed in 24-well polystyrene microtiter plates were determined by counting the number of colony-forming units (CFUs), as previously described (Zheng et al., 2019). To investigate whether RZD or LZD could inhibit *E. faecalis* biofilm formation, *E. faecalis* isolates were inoculated into 96-well polystyrene microtiter plates with TSBG containing RZD or LZD (at sub-MICs). After 24 h of static incubation, biofilm biomass was determined by crystal violet staining. Each assay was performed in triplicate at least three times.

### RT-qPCR to Determine the RNA Levels of *E. faecalis* Biofilm Formation-Related Genes

The RNA levels of 16 biofilm formation-related genes of the above 13 *E. faecalis* clinical isolates were determined by RT-qPCR based on published reports (Tendolkar et al., 2005; Nallapareddy et al., 2006; Guiton et al., 2009; Chavez de Paz et al., 2012; Soares et al., 2014; Dale et al., 2015; Frank et al., 2015; Akbari Aghdam et al., 2017; Zheng et al., 2018). The *E. faecalis* clinical isolates were inoculated into 100 mm × 20 mm non-pyrogenic polystyrene cell culture dishes with TSBG containing RZD or LZD (at 1/4× the MIC). After 6, 12, or 24 h of static incubation, total RNA was extracted from planktonic and biofilm *E. faecalis* cells for RT-qPCR. The primers used for RT-qPCR are listed in Supplementary Table S2. Each assay was performed in triplicate at least three times.

### Statistical Analysis

The data were analyzed using the Student’s *t*-test. *P-*values < 0.05 were considered significant. All data were analyzed in SPSS version 16.0 (SPSS, Inc., Chicago, IL, United States).

## Results

### RZD Was More Effective Than LZD Against *E. faecalis* Clinical Isolates

The distribution of MICs for RZD and LZD and their relationship with the susceptibilities for some other conventional antimicrobials are shown in Table 1. The data show that the MIC_50_/MIC_90_ of RZD was eightfold lower than that of LZD, suggesting that the *in vitro* activity of RZD against the 302 *E. faecalis* clinical isolates was substantially higher than that of LZD. Moreover, the MICs of RZD against the 52 LZD-non-susceptible *E. faecalis* clinical isolates were also lower (Supplementary Table S5). Twenty-one LZD-non-susceptible clinical isolates presented mutations in domain V of the 23S rRNA gene, but only four isolates contained the *optrA* gene. As shown in Supplementary Table S5, only two of the four *optrA*-carrying isolates (16C112 and 16C154) harbored mutations in the V domain of the 23S rRNA gene. No mutations were detected in ribosomes L3 and L4, or in the *cfr*, *cfr(B)*, *poxtA* genes in these isolates (data not shown).

**TABLE 1:** The distribution of radezolid and linezolid minimum inhibitory concentrations (MICs) in 302 *Enterococcus faecalis* clinical isolates.

Antimicrobials	RZD MIC distribution (mg/L)					LZD MIC distribution (mg/L)				
		
	≤ 0.125	0.25	0.5	≥ 1	*MIC_50_/MIC_90_*	≤ 1	2	4	≥ 8	*MIC_50_/MIC_90_*
**Ampicillin**										
**S**	83	163	53	1	*0.25/0.5*	80	168	42	10	*2/4*
**R**	1	1	0	0	≤*0.125/0.25*	1	1	0	0	*1/2*
**Doxycycline**										
**S/I**	19	35	11	0	*0.25/0.5*	23	35	6	1	*2/2*
**R**	66	128	42	1	*0.25/0.5*	57	135	36	9	*2/4*
**Vancomycin**										
**S/I**	85	163	53	1	*0.25/0.5*	80	170	42	10	*2/4*
**R**	0	0	0	0	–	0	0	0	0	–
**Erythromycin**										
**S/I**	22	31	9	0	*0.25/0.5*	10	42	10	0	*2/4*
**R**	63	132	44	1	*0.25/0.5*	70	128	32	10	*2/4*
**Ciprofloxacin**										
**S/I**	64	129	37	1	*0.25/0.5*	60	140	26	5	*2/4*
**R**	21	33	17	0	*0.25/0.5*	19	31	16	5	*2/4*
**Nitrofurantoin**										
**S/I**	83	162	53	1	*0.25/0.5*	77	170	42	10	*2/4*
**R**	2	1	0	0	≤*0.125/0.25*	3	0	0	0	*1/1*
**Rifampin**										
**S/I**	15	25	8	1	*0.25/0.5*	13	25	9	2	*2/4*
**R**	70	138	45	0	*0.25/0.5*	67	145	33	8	*2/4*
**Amikacin**										
**S/I**	17	27	7	1	*0.25/0.5*	18	23	9	2	*2/4*
**R**	67	137	46	0	*0.25/0.5*	61	148	33	8	*2/4*

*RZD, radezolid; LZD, linezolid; S, sensitive; I, intermediate; R, resistant.*

### Effects of RZD on High-Level LZD-Resistant *E. faecalis* Isolates

To evaluate the effect of RZD on the high-level LZD-resistant *E. faecalis* isolates and explore the extent to which RZD overcame LZD-related resistance mechanisms, high-level resistance to LZD was induced in the *E. faecalis* ATCC29212 and OG1RF strains. Table 2 shows the primary mutation loci in the 23S rRNA gene in the high-level LZD-resistant G2576U isolates. Mutations in ribosomes L3 and L4, and in the *cfr*, *cfr(B)*, *optrA*, and *poxtA* genes were not detected in these isolates (data not shown). The MICs for RZD increased sharply to ≥ 4 mg/L if the isolates presented with LZD-induced mutations in the four copies of domain V of the 23S rRNA gene (linezolid MICs ≥ 64 mg/L).

**TABLE 2:** High-level linezolid resistance leads to the decreased sensitivity of *Enterococcus faecalis* to radezolid.

Isolates	MIC (mg/L)		23S rRNA mutations			
		
	LZD	RZD	R1	R2	R3	R4
ATCC29212-0	2	0.25	–	–	–	–
ATCC29212-6	8	0.25	C2424U	–	–	G2576U
ATCC29212-10	16	0.5	C2424U	–	–	G2576U
ATCC29212-18	64	1	C2424U	–	G2576U	G2576U
ATCC29212-22	128	1	C2424U	–	G2576U	G2576U
ATCC29212-27	256	4	C2424U	G2576U	G2576U	G2576U
ATCC29212-47	256	8	C2424U	G2576U	G2576U	G2576U
OG1RF-0	2	0.25	–	–	–	–
OG1RF-5	8	0.25	G2576U	–	G2505A	–
OG1RF-8	16	1	G2576U	–	G2505A	G2576U
OG1RF-18	64	4	G2576U, C2610A	G2576U, C2610A	G2505A	G2576U, C2610A
OG1RF-25	128	4	G2576U, C2610A	G2576U, C2610A	G2505A	G2576U, C2610A
OG1RF-30	256	4	G2576U, C2610A	G2576U, C2610A	G2505A	G2576U, C2610A
OG1RF-55	256	8	G2576U, C2610A	G2576U, C2610A	G2505A	G2576U, C2610A

*RZD, radezolid; LZD, linezolid; MIC, minimum inhibitory concentration.*

### Efflux Pumps Are Involved in *E. faecalis* Resistance to RZD and LZD

**FIGURE 1:**
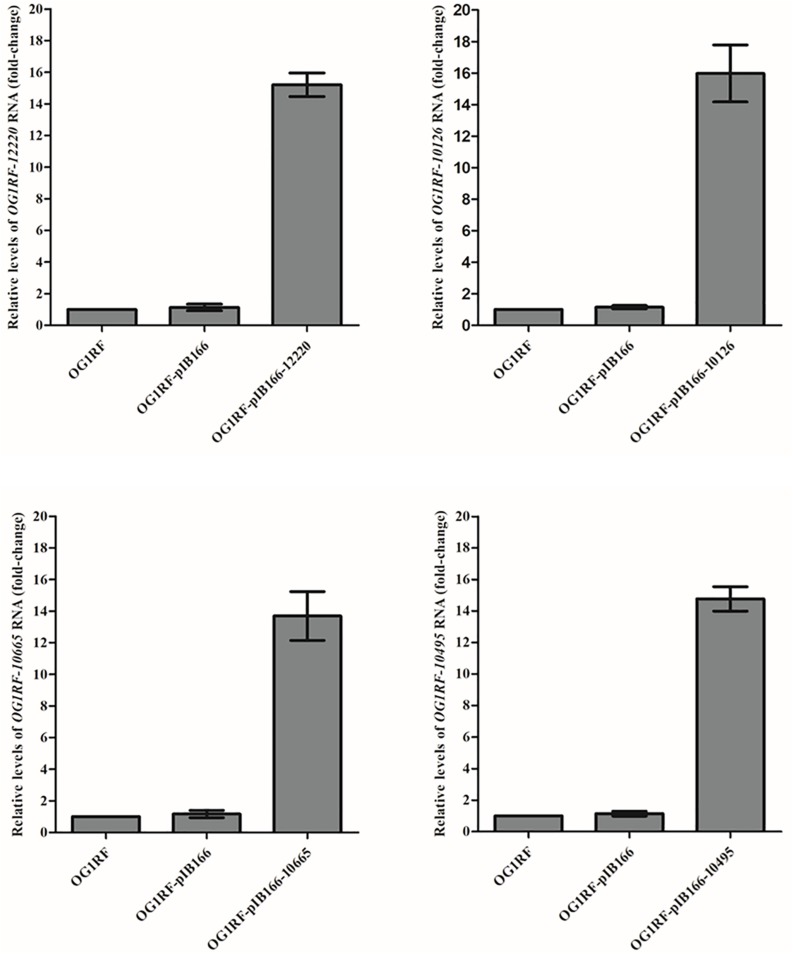
Overexpression of *OG1RF_12220, OG1RF_10126, OG1RF_10665*, and *OG1RF_10495* in the *Enterococcus faecalis* OG1RF strain. The RNA levels of the four genes were determined by RT-qPCR. The OG1RF wild-type isolate was used as the reference strain (mRNA level = 1.0). The OG1RF isolate containing the empty pIB166 vector (OG1RF-pIB166) was used as control.

The MICs for LZD or RZD showed a two- to eight-fold decrease in the presence of PAβN (Table 3). Thus, to explore the role of efflux pumps in RZD and LZD resistance, we assessed the changes occurring in the transcriptome of the high-level LZD-resistant OG1RF isolate (OG1RF-55, linezolid MIC: 256 mg/L; radezolid MIC: 8 mg/L) by RNA-seq (Supplementary Figure S1 and Supplementary Table S6). Subsequently, we evaluated the RNA expression levels of efflux pump-related genes by RT-qPCR, and found that the transcriptional levels of four ABC superfamily ATP-binding cassette transporter genes (*OG1RF_12220, OG1RF_10126, OG1RF_10665*, and *OG1RF_10495*) were increased in the high-level LZD-resistant OG1RF-55 isolate (Table 4). Finally, to confirm that the four genes were involved in the resistance to RZD and LZD, we overexpressed these genes in the LZD- and RZD-sensitive OG1RF isolate (Figure 1 and Supplementary Tables S3, S4). As indicated in Table 5, the MICs for LZD and RZD increased only with overexpression of *OG1RF_12220* (*mdlB2*).

**TABLE 3:** Radezolid and linezolid minimum inhibitory concentrations (MICs) decreased in the presence of PAβN.

Isolates	MIC (mg/L)			
	
	LZD	LZD + PAβN	RZD	RZD + PAβN
ATCC29212-0	2	2	0.25	0.25
ATCC29212-6	8	4	0.25	0.25
ATCC29212-10	16	4	0.5	0.25
ATCC29212-18	64	8	1	0.25
ATCC29212-22	128	32	1	0.5
ATCC29212-27	256	32	1	0.5
ATCC29212-47	256	64	8	2
OG1RF-0	2	2	0.25	0.25
OG1RF-5	8	2	0.25	0.25
OG1RF-8	16	4	1	0.25
OG1RF-18	64	8	4	0.5
OG1RF-25	128	16	4	1
OG1RF-30	256	16	4	1
OG1RF-55	256	32	8	2

*RZD, radezolid; LZD, linezolid; PAβN, Phe-Arg-β-naphthylamide (20 mg/L).*

**TABLE 4:** Differential RNA levels of efflux pump-related genes in a high-level linezolid-resistant isolate.

Gene_ID	Gene locus_tag (gene name)	Function/description	RNA levels of OG1RF-55/OG1RF-0	
			
			RNA-seq	RT-qPCR
*OG1RF_RS11380*	*OG1RF_12220 (mdlB2)*	Multidrug ABC superfamily ATP-binding cassette transporter, ABC protein	9.046	8.073
*OG1RF_RS00635*	*OG1RF_10126*	ABC superfamily ATP-binding cassette transporter, ABC protein	6.334	4.813
*OG1RF_RS03455*	*OG1RF_10665*	ABC superfamily ATP-binding cassette transporter, ABC protein	5.449	6.538
*OG1RF_RS02620*	*OG1RF_10495*	ABC superfamily ATP-binding cassette transporter, ABC protein	4.349	3.452
*OG1RF_RS13115*	*OG1RF_12562 (oadA)*	Oxaloacetate decarboxylase	3.422	1.698
*OG1RF_RS00860*	*OG1RF_10171 (secY)*	Preprotein translocase subunit SecY	2.998	0.913
*OG1RF_RS07425*	*OG1RF_11442 (mdlB)*	Multidrug ABC superfamily ATP-binding cassette transporter, ABC protein	2.678	1.544
*OG1RF_RS07430*	*OG1RF_11443 (mdlA)*	Multidrug ABC superfamily ATP-binding cassette transporter, ABC protein	2.595	1.948
*OG1RF_RS03320*	*OG1RF_10638 (oppD)*	ABC superfamily ATP-binding cassette transporter, ABC protein	2.537	1.478
*OG1RF_RS08840*	*OG1RF_11726*	ABC superfamily ATP-binding cassette transporter, ABC protein	2.532	1.582
*OG1RF_RS04005*	*OG1RF_10775*	Drug: H + antiporter-1 family protein	2.383	1.841
*OG1RF_RS04550*	*OG1RF_10869*	von Willebrand factor type A domain protein	2.331	1.745
*OG1RF_RS05865*	*OG1RF_11131*	ABC superfamily ATP-binding cassette transporter, ABC protein	2.296	2.205
*OG1RF_RS11385*	*OG1RF_12221*	ABC superfamily ATP-binding cassette transporter, ABC/membrane protein	2.145	1.672
*OG1RF_RS04555*	*OG1RF_10870*	Cell wall surface anchor family protein	2.127	1.021
*OG1RF_RS03325*	*OG1RF_10639 (oppF)*	ABC superfamily ATP-binding cassette transporter, ABC protein	2.004	1.969
*OG1RF_RS05110*	*OG1RF_10982*	Response regulator	0.357	0.527
*OG1RF_RS03235*	*OG1RF_10620*	ABC superfamily ATP-binding cassette transporter, ABC protein	0.222	0.369
*OG1RF_RS11320*	*OG1RF_12207*	ABC superfamily ATP-binding cassette transporter, ABC protein	0.200	0.047

*The *Enterococcus faecalis* OG1RF wild-type isolate (OG1RF-0, linezolid- and radezolid-sensitive) and the high-level linezolid-resistant isolate (OG1RF-55, linezolid MIC: 256 mg/L; radezolid MIC: 8 mg/L) were inoculated and grown to the logarithmic phase (4 h), total RNA was isolated and sequenced by Illumina HiSeq 2500 sequencer. Data are the means of the results from three independent experiments.*

**TABLE 5:** Radezolid and linezolid minimum inhibitory concentrations (MICs) increased with *OG1RF_12220* overexpression in the *Enterococcus faecalis* OG1RF strain.

Strains	MIC (mg/L)	
	
	Linezolid	Radezolid
OG1RF	2	0.25
OG1RF-*12220*	4	0.5
OG1RF-*10126*	2	0.25
OG1RF-*10665*	2	0.25
OG1RF-*10495*	2	0.25

### RZD Inhibited *E. faecalis* Biofilm Formation to a Greater Extent Than LZD

**FIGURE 2:**
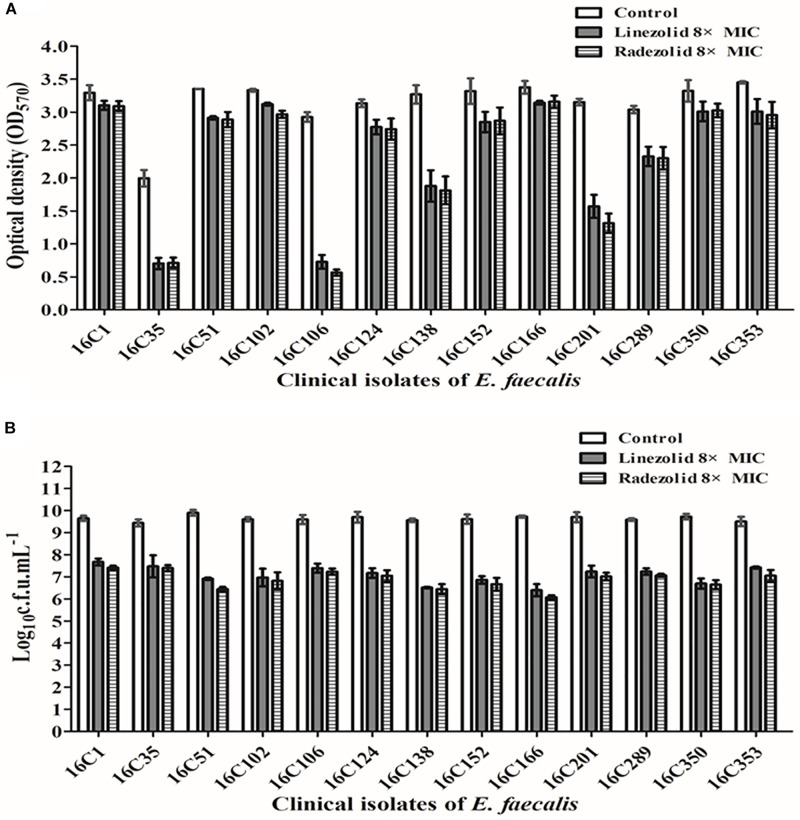
Radezolid (RZD) and linezolid (LZD) eradicated established biofilms and biofilm adherent cells of *E. faecalis*. The 13 *E. faecalis* clinical isolates were allowed to form mature biofilms for 24 h, following which the established biofilms were treated with RZD or LZD (at 8× their minimum inhibitory concentrations [MICs]) for 48 h. The remaining biofilm biomass was determined by crystal violet staining **(A)**; the adherent cells remaining in the biofilms were determined by the numbers of colony-forming units (CFUs) **(B)**. Data represent the average of three independent experiments (mean ± SD).

**FIGURE 3:**
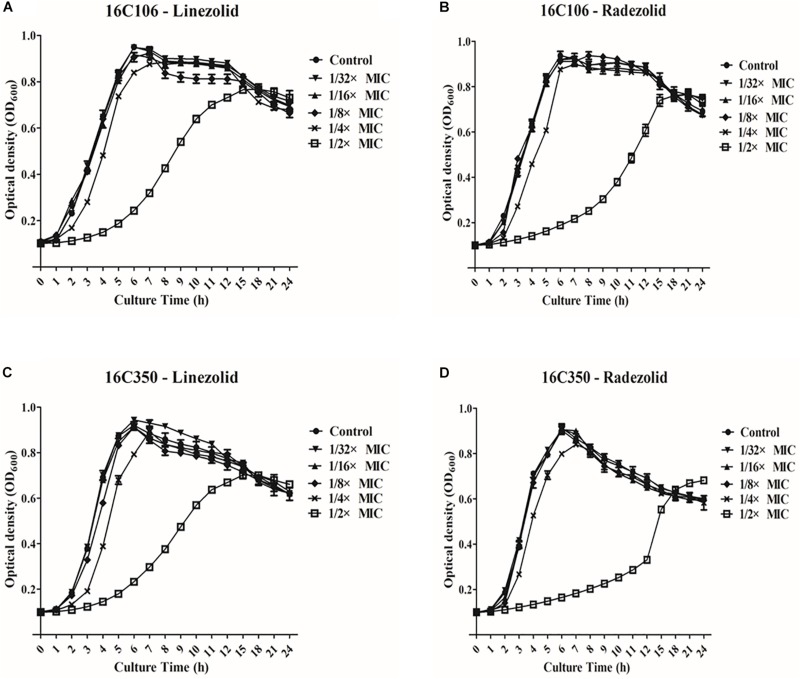
Planktonic growth of *E. faecalis* 16C106 and 16C350 isolates with radezolid (RZD) or linezolid (LZD) treatment. The growth of planktonic *E. faecalis* 16C106 cells treated with LZD **(A)** or RZD **(B)** and that of planktonic 16C350 cells treated with LZD **(C)** or RZD **(D)** was determined by measurement of the optical density at 600 nm (OD_600_). Data represent the average of three independent experiments (mean ± SD).

**FIGURE 4:**
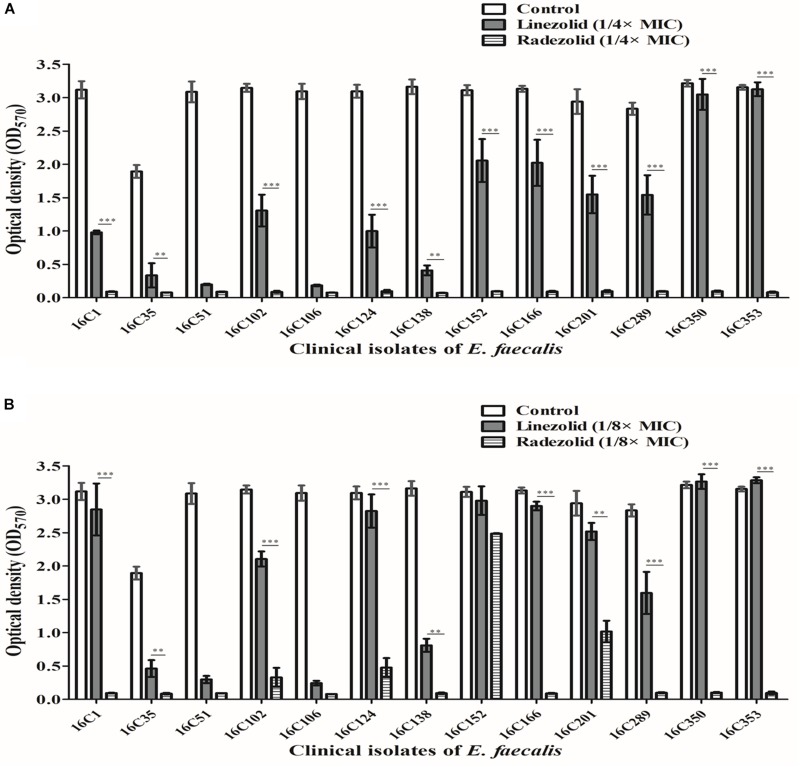
Sub-minimum inhibitory concentrations (MICs) (1/4 or 1/8×) of radezolid (RZD) and linezolid (LZD) inhibited *E. faecalis* biofilm formation. The 13 *E. faecalis* clinical isolates were treated with RZD or LZD at 1/4× **(A)** or 1/8× **(B)** their MICs for 24 h, and then biofilm biomass was determined by crystal violet staining. Data represent the average of three independent experiments (mean ± SD). **P < 0.01, ***P < 0.001 (Student’s *t*-test).

**FIGURE 5:**
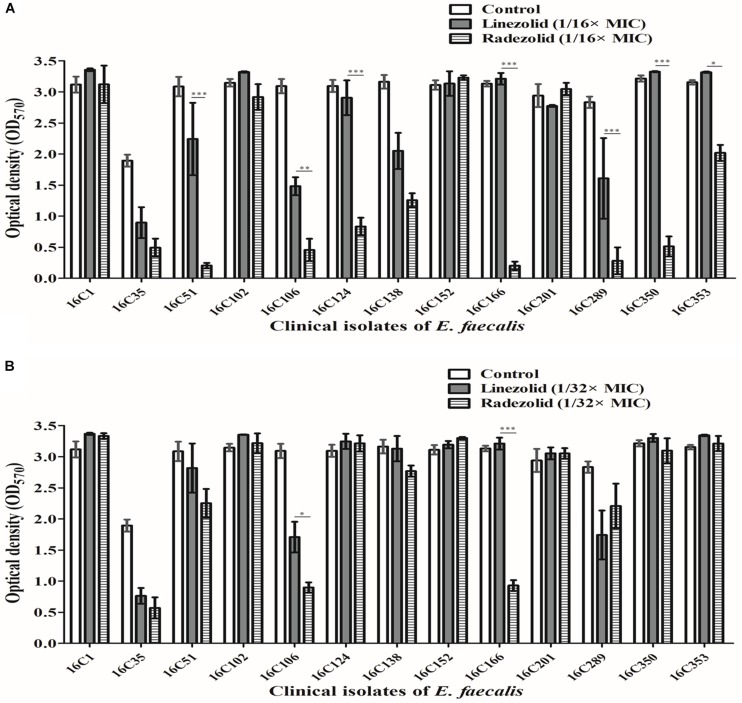
Sub-minimum inhibitory concentrations (MICs) (1/16 or 1/32×) of radezolid (RZD) and linezolid (LZD) inhibited *E. faecalis* biofilm formation. The 13 *E. faecalis* clinical isolates were treated with RZD or LZD at 1/16× **(A)** or 1/32× **(B)** their MICs for 24 h, and then biofilm biomass was determined by crystal violet staining. Data represent the average of three independent experiments (mean ± SD). *P < 0.05, **P < 0.01, ***P < 0.001 (Student’s *t*-test).

Thirteen *E. faecalis* clinical isolates (biofilm-positive) were selected to compare the differential effects of RZD and LZD on *E. faecalis* biofilms (Supplementary Table S7). First, we compared the eradicating potential of RZD and LZD (at 8× their MICs) on established *E. faecalis* biofilms, and found no difference between them (Figure 2). Both drugs also elicited similar effects on adherent cells of established biofilms. We also compared the sub-MICs at which RZD and LZD inhibited *E. faecalis* biofilm formation. Based on a previous study (Mlynek et al., 2016) and our preliminary results, 1/4, 1/8, 1/16, and 1/32× MICs were used in this study. The 1/2× MIC was not used because the planktonic growth of *E. faecalis* was markedly affected under this concentration of both RZD and LZD (Figure 3). As shown in Figure 4, the RZD at 1/4 or 1/8× its MIC efficiently inhibited *E. faecalis* biofilm formation and to a greater extent than LZD. This trend was also observed in several of the 13 clinical isolates at MICs of 1/16 or 1/32× (Figure 5).

### RZD Treatment Reduced the RNA Levels of Biofilm Formation-Related Genes

Isolates 16C106 and 16C350 were selected for RT-qPCR to evaluate the RNA expression levels of 16 *E. faecalis* biofilm formation-related genes at different stages of biofilm formation. The RNA levels of *ahrC*, *cylA*, *esp*, *relA*, and *relQ* markedly decreased when the isolates were treated with RZD or LZD at 1/4× their MICs for 6 h (Table 6). The RNA levels of these 16 genes in the other 11 *E. faecalis* clinical isolates were also determined, and, as indicated in Table 7, the transcriptional levels of *ahrC*, *esp*, *relA*, and *relQ* showed a significant decrease, especially in isolates treated with RZD.

**TABLE 6:** Changes in the RNA expression levels of biofilm formation-related genes of isolates 16C106 and 16C350 with radezolid or linezolid treatment.

Biofilm formation-related genes	16C106							16C350				
		
	Radezolid			Linezolid			Radezolid			Linezolid		
				
	6 h	12 h	24 h	6 h	12 h	24 h	6 h	12 h	24 h	6 h	12 h	24 h
*Agg*	0.457	0.793	1.373	0.633	0.916	1.577	–	–	–	–	–	–
*ahrC*	0.194	0.694	2.658	0.211	1.401	2.217	0.110^c^	0.603	1.434	0.421	1.655	1.779
*asa1*	0.662	1.256	2.662	0.778	1.764	2.178	1.528	1.413	1.850	0.800	1.056	1.455
*Atn*	1.065	1.351	2.572	1.490	2.073	1.818	2.212	1.332	1.766	1.613	2.509	1.429
*cylA*	0.186^a^	0.397	1.675	0.481	0.569	0.952	0.175^c^	0.415	0.839	0.516	1.567	1.180
*Eep*	–	–	–	–	–	–	0.943	2.068	0.536	1.735	5.138	1.485
*ebpA*	0.772	1.516	4.725	0.685	7.799	3.691	2.658	5.318	1.690	1.880	3.193	1.185
*epaI*	0.693	1.467	3.154	0.638	3.976	3.389	2.095	6.424	1.218	3.472	6.235	1.843
*epaOX*	0.859	2.729	4.933	1.035	1.662	4.324	2.939	4.336	0.943	2.669	4.403	1.765
*Esp*	0.089^b^	0.806	2.550	0.178	1.405	2.597	0.196^c^	0.993	1.456	0.517	2.896	1.562
*fsrA*	2.083	2.281	6.663	2.546	3.004	2.802	1.895	1.627	1.878	1.893	1.812	2.138
*gelE*	–	–	–	–	–	–	–	–	–	–	–	–
*hyl*	–	–	–	–	–	–	1.554	1.526	2.365	1.859	2.019	2.398
*relA*	0.103^b^	0.567	1.966	0.246	1.565	3.965	0.218^d^	2.528	2.385	0.520	2.299	3.269
*relQ*	0.182^b^	0.624	3.098	0.314	1.487	4.562	0.241^c^	2.839	2.260	0.611	4.613	4.400
*srtA*	0.687	1.660	3.819	0.726	1.724	3.755	0.572	2.573	4.572	0.895	3.276	4.295

*Radezolid and linezolid were used at 1/4× their minimum inhibitory concentrations (MICs). RNA levels were detected by RT-qPCR, with untreated isolate as the reference strain (mRNA level = 1.0). −, not detected. ^*a*^16C106: Radezolid (6 h) vs. Linezolid (6 h), *P* < 0.01; ^*b*^16C106: Radezolid (6 h) vs. Linezolid (6 h), *P* < 0.05; ^*c*^16C350: Radezolid (6 h) vs. Linezolid (6 h), *P* < 0.01; ^*d*^16C350: Radezolid (6 h) vs. Linezolid (6 h), *P* < 0.05.*

**TABLE 7:** Changes in RNA expression levels of biofilm formation-related genes in 11 clinical isolates with radezolid or linezolid treatment.

	Biofilm formation-related genes															
	
	*agg*	*ahrC*	*asa1*	*atn*	*cylA*	*eep*	*ebpA*	*epaI*	*epaOX*	*esp*	*fsrA*	*gelE*	*hyl*	*relA*	*relQ*	*srtA*
**16C1**																
RZD	0.578	0.108^a^	0.784	1.358	0.388^c^	–	0.864	0.897	1.062	0.113^d^	1.234	0.687	1.657	0.218^f^	0.359	0.687
LZD	0.864	0.368	0.895	1.295	0.664	–	1.234	1.065	1.364	0.268	1.498	0.892	1.552	0.458	0.487	0.734
**16C35**																
RZD	0.854	0.169^b^	1.285	1.069	0.295	0.965	0.937	1.298	1.118	0.165^d^	0.931	0.885	–	0.311^f^	0.365	1.058
LZD	1.068	0.293	1.154	1.035	0.431	1.262	1.364	1.105	0.997	0.378	0.854	1.158	–	0.524	0.487	1.364
**16C51**																
RZD	0.814	0.167	1.025	1.168	0.505	–	1.035	0.954	1.548	0.075^d^	0.875	–	–	0.309^f^	0.224	0.564
LZD	0.962	0.208	1.132	0.864	0.598	–	1.125	1.264	1.068	0.119	0.965	–	–	0.524	0.368	0.635
**16C102**																
RZD	–	0.263^b^	–	0.864	–	0.597	0.824	0.954	0.931	0.138^d^	0.768	0.687	–	0.269^f^	0.218^h^	0.694
LZD	–	0.597	–	1.126	–	0.854	0.789	1.158	0.865	0.367	0.895	0.885	–	0.486	0.531	0.954
**16C124**																
RZD	–	0.597	1.164	1.106	0.167^c^	0.364	0.764	0.854	0.954	0.107^e^	0.964	0.989	–	0.208	0.497	1.065
LZD	–	0.631	0.965	1.357	0.284	0.543	0.805	0.762	1.035	0.485	1.165	1.164	–	0.294	0.551	1.321
**16C138**																
RZD	–	0.156	0.881	1.265	0.464	–	1.068	0.924	1.268	0.255^d^	1.123	–	–	0.368^f^	0.305^h^	1.246
LZD	–	0.198	1.098	1.067	0.657	–	1.267	1.157	0.954	0.495	1.065	–	–	0.542	0.687	1.098
**16C152**																
RZD	0.954	0.468	1.065	1.196	0.365^c^	1.354	0.854	1.094	1.354	0.073^e^	0.882	0.724	–	0.097^g^	0.267^h^	0.769
LZD	1.367	0.598	1.298	0.932	0.697	1.158	1.063	1.265	1.165	0.396	0.854	0.658	–	0.264	0.576	0.854
**16C166**																
RZD	–	0.097^b^	0.854	1.267	–	0.862	0.962	1.164	1.264	–	0.931	–	–	0.168^f^	0.186^h^	0.367
LZD	–	0.167	0.801	1.065	–	1.065	1.264	0.938	1.129	–	1.095	–	–	0.291	0.367	0.431
**16C201**																
RZD	–	0.157^b^	0.652	0.597	–	–	0.531	0.789	0.631	–	0.934	–	–	0.103^g^	0.158	0.687
LZD	–	0.269	0.896	0.786	–	–	0.764	0.894	0.835	–	1.264	–	–	0.324	0.263	1.357
**16C289**																
RZD	0.764	0.298	1.068	1.267	0.368_*c*_	0.714	0.965	1.158	0.958	0.085^e^	0.867	0.534	2.674	0.208^f^	0.296^h^	0.974
LZD	0.878	0.367	1.165	1.152	0.587	1.257	1.247	1.036	1.168	0.298	0.906	0.738	1.687	0.593	0.687	1.265
**16C353**																
RZD	–	0.394	1.267	0.954	0.464	–	0.854	0.964	1.068	0.069^e^	0.768	–	–	0.082^g^	0.158^i^	0.875
LZD	–	0.485	1.597	1.264	0.568	–	0.834	1.167	0.915	0.468	0.934	–	–	0.368	0.497	1.068

*RZD, radezolid; LZD, linezolid. Radezolid and linezolid were used at 1/4× their minimum inhibitory concentrations (MICs). The RNA levels were detected by RT-qPCR, with untreated isolate as the reference strain (mRNA level = 1.0). −, not detected. ^*a*^*ahrC*: RZD vs. LZD, *P* < 0.01; ^*b*^*ahrC*: RZD vs. LZD, *P* < 0.05; ^*c*^*cylA*: RZD vs. LZD, *P* < 0.05; ^*d*^*esp*: RZD vs. LZD, *P* < 0.05; ^*e*^*esp*: RZD vs. LZD, *P* < 0.01; ^*f*^*relA*: RZD vs. LZD, *P* < 0.05; ^*g*^*relA*: RZD vs. LZD, *P* < 0.01; ^*h*^*relQ*: RZD vs. LZD, *P* < 0.05; ^*i*^*relQ*: RZD vs. LZD, *P* < 0.01.*

## Discussion

Radezolid, a novel biaryl analog of LZD, exhibits excellent activity against gram-positive bacteria, including methicillin-resistant *Staphylococcus aureus* (MRSA) and LZD-resistant staphylococci (Lemaire et al., 2010a; Locke et al., 2010). The results of this study further indicate that RZD also exerts stronger effects than LZD against several *E. faecalis* clinical isolates. One study demonstrated that, in LZD-resistant *S. aureus* isolates (linezolid MICs ranging from 8 to 32 mg/L), the MICs of RZD against these isolates were two- to eight-fold lower (from 1 to 4 mg/L) than those of LZD (Locke et al., 2010). In this study, we also focused on LZD-non-susceptible *E. faecalis* clinical isolates (linezolid MICs ranging from 4 to 32 mg/L); however, we found that the MICs of RZD for these isolates (from 0.25 to 1 mg/L) were 8- to 32-fold lower than those of LZD. We also showed that when high-level resistance to LZD was induced in *E. faecalis* (linezolid MICs ≥ 64 mg/L), the MICs of RZD increased from 1 to 8 mg/L. This result indicated that cross-resistance to RZD and LZD was emerging in these isolates, and that this was mainly due to mutations in the four copies of domain V of the 23S rRNA gene.

The oxazolidinone class of antimicrobials, which includes LZD, exhibited excellent effects against gram-positive bacteria, but showed poor activity against gram-negative bacteria such as *Escherichia coli*, primarily due to the enhanced activity of AcrB, a RND-type efflux pump (Schumacher et al., 2007; Schuster et al., 2019). However, whether efflux pumps have a role in the resistance of gram-positive bacteria to LZD or RZD remains uncertain. In the present study, we found that the MICs of LZD and RZD decreased in the presence of PAβN, an efflux pump inhibitor, indicating that efflux pumps are involved in *E. faecalis* resistance to LZD and RZD. We also found that the enhanced activity of *OG1RF-12220* (*mdlB2*) led to increases in the MICs of both LZD and RZD. The expression of the efflux pump gene *mdlB* was reported to be substantially increased among fluoroquinolone-resistant isolates of *Salmonella enterica* (Chen et al., 2007). Both *mdlB* and *mdlB2* belong to the efflux transport system of the ATP-binding cassette (ABC) superfamily, which plays an important role in antimicrobial resistance (Iannelli et al., 2018). We found that *mdlB2*, but not *mdlB*, was involved in the resistance to LZD and RZD. However, this result requires further experimental confirmation, especially in high-level LZD-resistant *E. faecalis* clinical isolates.

Several studies have indicated that LZD affects the biofilms of *E. faecalis*, both when administered alone or in combination with rifampicin or gentamicin (Bayston et al., 2012; Holmberg et al., 2012; Luther et al., 2014). In this study, we found that RZD had a significantly greater effect than LZD on planktonic *E. faecalis* cells. We further investigated whether this was also true for *E. faecalis* biofilm formation, but we did not find any difference between RZD and LZD in eradicating established biofilms or adherent cells in the biofilms of *E. faecalis*. Interestingly, we found that RZD (at 1/4 or 1/8× the MIC) strongly inhibited *E. faecalis* biofilm formation, and more effectively than LZD. This result was similar to that of a previous study, in which Wu et al. (2014) found that another oxazolidinone, FYL-67, could more strongly inhibit *S. aureus* biofilm formation than LZD.

In the present study, we also explored the reasons for the RZD-mediated reduction in *E. faecalis* biofilm formation, and found that the mRNA levels of *ahrC*, *esp*, *relA*, and *relQ* were significantly decreased with RZD treatment. Several studies have demonstrated that the Esp virulence factor, which has been found to support *E. faecalis* cell adherence, colonization, and persistence in the urinary tract, also plays an important role in *E. faecalis* biofilm formation (Toledo-Arana et al., 2001; Tendolkar et al., 2004; Zheng et al., 2017). The *ahrC* gene, which encodes a transcriptional regulator of the ArgR family, was found to be critical for *E. faecalis* attachment to polystyrene *in vitro*, and porcine heart valve surfaces *ex vivo*, during the early stages of biofilm formation (Frank et al., 2013). The hydrolase RelA and the small alarmone synthetase RelQ, encoded by the *relA* and *relQ* genes, respectively, have also been found to control (p)ppGpp metabolism and sustain biofilm formation in *E. faecalis* (Chavez de Paz et al., 2012). Our study indicated that the transcriptional levels of *ahrC*, *esp*, *relA*, and *relQ* were greatly decreased when *E. faecalis* clinical isolates were treated with RZD for 6 h, resulting in a significant reduction in biofilm formation by *E. faecalis*.

## Conclusion

We showed that radezolid was more effective than linezolid against planktonic *E. faecalis* cells. Additionally, this study is the first to report that the *mdlB2* gene is important for *E. faecalis* resistance to both RZD and LZD. We also found that RZD was more effective than LZD at inhibiting *E. faecalis* biofilm formation, which was mainly achieved through inhibition of the transcription of *ahrC*, *esp*, *relA* and *relQ* in *E. faecalis*.

## Data Availability Statement

The datasets generated for this study can be found in the Sequence Read Archive (SRA) database under accession number PRJNA505107 (https://www.ncbi.nlm.nih.gov/bioproject/PRJNA505107).

## Ethics Statement

All procedures involving human participants were performed in accordance with the ethical standards of Shenzhen University School of Medicine and with the 1964 Helsinki Declaration and its later amendments, and this study was approved by the Ethics Committee of the Shenzhen University School of Medicine. For this type of study, formal consent is not required.

## Author Contributions

JZ designed the study, performed gene manipulation, analyzed and interpreted the RNA-seq data, performed the biofilm assay, and drafted the manuscript. ZC performed gene manipulation, MIC detection, mRNA extraction, and RNA-seq and RT-qPCR data analysis. ZL conducted the LZD *in vitro* induction, MIC detection, and analyses of RNA-seq and overexpression data. XS performed MIC detection, gene manipulation, RT-qPCR, biofilm assay, and bacteria counting in the biofilm assay. BB, GX, and JC performed MIC detection, LZD *in vitro* induction, RT-qPCR, biofilm assay, and bacteria counting in the biofilm assay. ZY and DQ designed the study, analyzed the data, and critically revised the manuscript for important intellectual content.

## Conflict of Interest

The authors declare that the research was conducted in the absence of any commercial or financial relationships that could be construed as a potential conflict of interest.

## Supplementary Material

FIGURE S1 The volcano plot of all the genes measured by RNA-seq.

TABLE S1 PCR primers used for the detection of linezolid resistance genes.

TABLE S2 RT-qPCR primers used for the detection of the RNA levels of the efflux pump-related genes and the biofilm formation-related genes.

TABLE S3 Strains and plasmids used for the overexpression of *OG1RF_12220, OG1RF_10126, OG1RF_10665* and *OG1RF_10495*, in *E. faecalis* OG1RF strain.

TABLE S4 PCR primers used for the overexpression of *OG1RF_12220, OG1RF_10126, OG1RF_10665*, and *OG1RF_10495* in *E. faecalis* OG1RF strain.

TABLE S5 Radezolid against 52 linezolid-nonsusceptible *E. faecalis* clinical isolates.

TABLE S6 Differential RNA levels of the genes in a high-level linezolid-resistant isolate.

TABLE S7 Radezolid and linezolid minimum inhibitory concentrations (MICs)in 13 biofilm positive *E. faecalis* clinical isolates.
