# Supplementary figures and images for: Radezolid Is More Effective Than Linezolid Against Planktonic Cells and Inhibits Enterococcus faecalis Biofilm Formation
Source: Front Microbiol. 2020 Feb 14;11:196. doi: 10.3389/fmicb.2020.00196 (PMC7033516; doi:10.3389/fmicb.2020.00196)

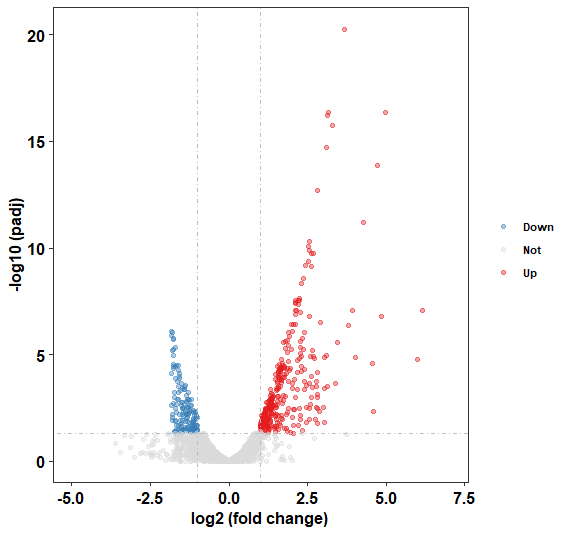

Supplement: FIGURE S1 — The volcano plot of all the genes measured by RNA-seq. [file Image_1.tiff]
