# Supplementary material for: Radezolid Is More Effective Than Linezolid Against Planktonic Cells and Inhibits Enterococcus faecalis Biofilm Formation
Source: Front Microbiol. 2020 Feb 14;11:196. doi: 10.3389/fmicb.2020.00196 (PMC7033516; doi:10.3389/fmicb.2020.00196)
Supplement: TABLE S1 — PCR primers used for the detection of linezolid resistance genes. [file Table_1.DOCX]

**TABLE S1︱** PCR primers used for the detection of linezolid resistance genes

| **Target genes** | **Primers** | **Sequences (5' → 3')** | **Reference** |
| --- | --- | --- | --- |
| 23S rRNA | 23S-F | GGCGCTGGTGGGATACTA | Bourgeois-Nicolaos N, et al. J Infect Dis 2007;195(10):1480-1488 |
|  | 23S-1R | GGACGGTTATGAGCCGTC |  |
|  | 23S-F | GGCGCTGGTGGGATACTA |  |
|  | 23S-2R | GCGATCTCCTGCGTGAC |  |
|  | 23S-F | GGCGCTGGTGGGATACTA |  |
|  | 23S-3R | CCCTTCTTCAAGCTTATC |  |
|  | 23S-F | GGCGCTGGTGGGATACTA |  |
|  | 23S-4R | CCACAGTGATTTTGCCCA |  |
|  | 23S-V/F | AGTTTGACTGGGGCGGTC |  |
|  | 23S-V/R | CCGGTCCTCTCTACTA |  |
| Ribosome L3 | *rplC*-F | ATGACCAAAGGAATCTTAGGG | Diaz L, et al. Antimicrob Agents Chemother 2012; 56(7):3917-3922 |
|  | *rplC*-R | CACAGCTGATTTGATWGTGATT |  |
| Ribosome L4 | *rplD*-F | GCCGAATGTAGCATTATTCAA |  |
|  | *rplD*-R | CAAGCACCTCCTCAATTTGAGT |  |
| *cfr* | *cfr*-F | TGTATGTTTTGACTTTCGGCACCGG AGT CA | Diaz L, et al. Antimicrob Agents Chemother 2012; 56(7):3917-3922 |
|  | *cfr*-R | ATTATCTTCCACCCAGTAGTCC |  |
| *cfr(B)* | *cfrB*-F | ATAACGGTTCTTCCTAAATCACTA A | Deshpande LM, et al. Antimicrob Agents Chemother 2015; 59(10):6256-6261 |
|  | *cfrB*-R | CATCTAATGTATCCATCACATCTG |  |
| *optrA* | *optrA*-F | AGGTGGTCAGCGAACTAA | Wang Y, et al. J Antimicrob Chemother 2015; 70(8):2182-2190 |
|  | *optrA*-R | ATCAACTGTTCCCATTCA | 70(8):2182-2190 |
| *poxtA* | *poxtA*-F | GGTGGATTTACCGACACCGT | Lei CW, et al. J Antimicrob Chemother. 2019;74(8):2459-2461 |
|  | *poxtA*-R | GACCAGTGGAAATGCCCGTA |  |
