# Supplementary material for: Radezolid Is More Effective Than Linezolid Against Planktonic Cells and Inhibits Enterococcus faecalis Biofilm Formation
Source: Front Microbiol. 2020 Feb 14;11:196. doi: 10.3389/fmicb.2020.00196 (PMC7033516; doi:10.3389/fmicb.2020.00196)
Supplement: TABLE S2 — RT-qPCR primers used for the detection of the RNA levels of the efflux pump-related genes and the biofilm formation-related genes. [file Table_2.DOCX]

**TABLE S2︱**RT-qPCR primers used for the detection of the RNA levels of the efflux pump-related genes and the biofilm formation-related genes

| **Primers** | **Sequences (5' →3')** | **Location (Genbank no.)** |
| --- | --- | --- |
| **Efflux pump related genes** |  |  |
| q12220-F | ATAAGAACCAACAAGGAA | 2340051-2340068 (CP002621.1) |
| q12220-R | AAGAACATCATCACTACC | 2339933-2339950 (CP002621.1) |
| q10126-F | ATCGTATCGCTGTTATTC | 140631-140648 (CP002621.1) |
| q10126-R | GCTTCTTCCAATGAGTAG | 140722-140739 (CP002621.1) |
| q10665-F | GATATTCATAGCGAACAAGTG | 700925-700945 (CP002621.1) |
| q10665-R | GAAGTCACGAGCATCTAC | 700793-700810 (CP002621.1) |
| q10495-F | GTCCTTCTGGTTCTGGTA | 515852-515869 (CP002621.1) |
| q10495-R | CTTGGTTGATGTCCGTATT | 515747-515765 (CP002621.1) |
| q12562-F | CCTGCCATATCCTTAATAC | 2726189-2726207 (CP002621.1) |
| q12562-R | CTTGAATGATGTCCGTAA | 2726351-2726368 (CP002621.1) |
| q10171-F | GTTGAATATGGTGAGTGGTA | 172980-172999 (CP002621.1) |
| q10171-R | AATAGAGGCTGTAATGTATGG | 173041-173061 (CP002621.1) |
| q11442-F | TTCCAGCAATATCTCCATAA | 1502431-1502450 (CP002621.1) |
| q11442-R | ATCGTGACATTCGTGATT | 1502509-1502526 (CP002621.1) |
| q11443-F | CCAGCACCAATCAAGAGT | 1505324-1505341 (CP002621.1) |
| q11443-R | GGCGTCGTTGTAGATGAA | 1505389-1505406 (CP002621.1) |
| q10638-F | CTGGAGATGCCTTCTATC | 677177-677194 (CP002621.1) |
| q10638-R | GCTTGTGGTGCTAATAAC | 677280-677297 (CP002621.1) |
| q11726-F | AAGCCACAGACATAATGATT | 1810905-1810924 (CP002621.1) |
| q11726-R | AATGGTAACGCAAGATGAA | 1811009-1811027 (CP002621.1) |
| q10775-F | TATACTGGCTGTTGATTA | 805558-805575 (CP002621.1) |
| q10775-R | CACCTGTTGATAATGTTC | 805672-805689 (CP002621.1) |
| q10869-F | ATAATAACAACGCCATTCAA | 907353-907372 (CP002621.1) |
| q10869-R | ACATATCACCAGCATCTC | 907461-907478 (CP002621.1) |
| q11131-F | TTCCAGGTGCTAATAGATTCA | 1175408-1175428 (CP002621.1) |
| q11131-R | ATTACCGACAGGCAACTT | 1175524-1175541 (CP002621.1) |
| q12221-F | AACTTACATTGCGGATGA | 2341085-2341102 (CP002621.1) |
| q12221-R | GAAGAAGGCGTAACAGAA | 2341173-2341190 (CP002621.1) |
| q10870-F | TGTCAGATAAGGCGGATAC | 910386-910404 (CP002621.1) |
| q10870-R | CGTCGTCAATGTGTAACC | 910466-910483 (CP002621.1) |
| q10639-F | AATGATATTATTGCTGAAGGAAT | 677712-677734 (CP002621.1) |
| q10639-R | CGTACTGTGACTTGGATT | 677811-677828 (CP002621.1) |
| q10982-F | ATTCACCAACCGACTATT | 1021402-1021419 (CP002621.1) |
| q10982-R | GCCGTAATCAGGATAACT | 1021560-1021577 (CP002621.1) |
| q10620-F | GTAACCACCACCATTGTC | 657398-657415 (CP002621.1) |
| q10620-R | GAACCAACTTCTGCCTTAG | 657474-657492 (CP002621.1) |
| q12207-F | CTATGAACTGAGCAACAA | 2327868-2327885 (CP002621.1) |
| q12207-R | GTAGGCATTACAACAATCT | 2328006-2328024 (CP002621.1) |
|  |  |  |
| **Biofilm formation related genes** |  |  |
| *agg*-F | CGTTGATAAAGCAGTTGAT | 52593-52611 (CP002494.1) |
| *agg*-R | TTGTAGTTGGTCTACTTCTT | 52482-52501 (CP002494.1) |
| *ahrC*-F | TTCCATTAGAAACACAAG | 741843-741860 (CP002621.1) |
| *ahrC*-R | GAGAACACTATCATCATC | 742022-742039 (CP002621.1) |
| *asa1*-F | CGCTATTACGAACTATGAC | 3208-3226 (X17214.1) |
| *asa1*-R | GACTTCCAGATACACAGA | 3383-3400 (X17214.1) |
| *atn*-F | AATAATCAATCAGGAACGAATACG | 760647-760670 (NC_004668.1) |
| *atn*-R | GCCACACTAACACCGAAT | 760718-760735 (NC_004668.1) |
| *cylA*-F | GGAGGATATGGTGACAAT | 934-951 (JQ794947.1) |
| *cylA*-R | TTACTTCTGGAGTTGCTAA | 1078-1096 (JQ794947.1) |
| *ebpA*-F | ATAATAACAACGCCATTCAA | 1058348- 1058367 (NC_004668.1) |
| *ebpA*-R | ACATATCACCAGCATCTC | 1058456-1058473 (NC_004668.1) |
| *eep*-F | AACAGATAGAGGCATACC | 1921320-1921337 (CP002621.1) |
| *eep*-R | GCACCACTTATACGATTC | 1921406-1921423 (CP002621.1) |
| *epaI*-F | AGCCGTTCCATCATATTG | 1813792-1813809 (CP002621.1) |
| *epaI*-R | ATGTGACTTCTGGTTATCG | 1813927- 1813945 (CP002621.1) |
| *epaOX*-F | CGTTGAGTAACATTATCGTATTG | 1794588-1794610 (CP002621.1) |
| *epaOX*-R | ATGAAGATATAGTGCCTACCT | 1794715-1794735 (CP002621.1) |
| *esp*-F | GCATCAGTATTAGTTGGT | 172-189 (AF034779.1) |
| *esp*-R | TTCCTTGTAACACATCAC | 350-367 (AF034779.1) |
| *fsrA*-F | GCCTGGATATGATTGTTC | 1591964- 1591981 (CP002621.1) |
| *fsrA*-R | CGTTAGAAGCATTGGTAA | 1592106-1592123 (CP002621.1) |
| *gelE*-F | TACACCATTATCCAGAACT | 1547-1565 (M37185.1) |
| *gelE*-R | CATCGCCATATTGAACTT | 1671-1688 (M37185.1) |
| *hyl*-F | CTTATCTTACCTTAACCAAT | 1336-1355 (AF544400.1) |
| *hyl*-R | CAATTCTGTTCTCAATCTA | 1493-1511 (AF544400.1) |
| *relA*-F | GGATAGATTGATACATATTCG | 1706210-1706230 (CP002621.1) |
| *relA*-R | TGACTTATTAGCCATTCG | 1706338-1706355 (CP002621.1) |
| *relQ*-F | TACGAACGATAGCCACTT | 2157245-2157262 (CP002621.1) |
| *relQ*-R | GCGGATTATGTGCCAATT | 2157356-2157373 (CP002621.1) |
| *srtA*-F | CGTGTTGAGTTAATTGATGA | 2930172-2930191 (NC_004668.1) |
| *srtA*-R | TTGCTGCTAATGTTCCTT | 2930263-2930280 (NC_004668.1) |
| recA-F**^a^** | CGACTAATGTCTCAAGCACTAC | 3044995-3045016 (NC_004668.1) |
| recA-R | CGAACATCACGCCAACTT | 3044911-3044928 (NC_004668.1) |

**^a^**: The internal control gene was recA (OG1RF_12439)
