# Supplementary material for: Radezolid Is More Effective Than Linezolid Against Planktonic Cells and Inhibits Enterococcus faecalis Biofilm Formation
Source: Front Microbiol. 2020 Feb 14;11:196. doi: 10.3389/fmicb.2020.00196 (PMC7033516; doi:10.3389/fmicb.2020.00196)
Supplement: TABLE S3 — Strains and plasmids used for the overexpression of OG1RF_12220, OG1RF_10126, OG1RF_10665 and OG1RF_10495, in E. faecalis OG1RF strain. [file Table_3.DOCX]

**TABLE S3︱**Strains and plasmids used for the overexpression of *OG1RF_12220, OG1RF_10126, OG1RF_10665* and *OG1RF_10495* in *E. faecalis* OG1RF strain

| **Strain or plasmid** | **Description** | **Source** |
| --- | --- | --- |
| ***E. faecalis* strains** |  |  |
| OG1RF | Rifampin- and fusidic acid-resistant derivative of human oral cavity isolate | ATCC |
| OG1RF-*12220* | OG1RF strain contained the plasmid pIB166-*OG1RF_12220* | This study |
| OG1RF-*10126* | OG1RF strain contained the plasmid pIB166-*OG1RF_10126* | This study |
| OG1RF-*10665* | OG1RF strain contained the plasmid pIB166-*OG1RF_10665* | This study |
| OG1RF-*10495* | OG1RF strain contained the plasmid pIB166-*OG1RF_10495* | This study |
| **Plasmids** |  |  |
| pIB166 | *E. coli* (Cm^20^)-*Streptococcus* (Cm^20^) shuttle vector | Jingren Zhang, Tsinghua University |
| pIB166-*OG1RF_12220* | pIB166 with insertion of the gene *OG1RF_12220*, for *OG1RF_12220* expression | This study |
| pIB166-*OG1RF_10126* | pIB166 with insertion of the gene *OG1RF_10126*, for *OG1RF_10126* expression | This study |
| pIB166-*OG1RF_10665* | pIB166 with insertion of the gene *OG1RF_10665*, for *OG1RF_10665* expression | This study |
| pIB166-*OG1RF_10495* | pIB166 with insertion of the gene *OG1RF_10495*, for *OG1RF_10495* expression | This study |
