# Supplementary material for: Radezolid Is More Effective Than Linezolid Against Planktonic Cells and Inhibits Enterococcus faecalis Biofilm Formation
Source: Front Microbiol. 2020 Feb 14;11:196. doi: 10.3389/fmicb.2020.00196 (PMC7033516; doi:10.3389/fmicb.2020.00196)
Supplement: TABLE S4 — PCR primers used for the overexpression of OG1RF_12220, OG1RF_10126, OG1RF_10665, and OG1RF_10495 in E. faecalis OG1RF strain. [file Table_4.DOCX]

**TABLE S4︱**PCR primers used for the overexpression of *OG1RF_12220, OG1RF_10126, OG1RF_10665* and *OG1RF_10495* in *E. faecalis* OG1RF strain

| **Primers** | **Sequence (5' → 3' )** | **Purpose** | **Annotation^a^** |
| --- | --- | --- | --- |
| 12220-F | CGCGGATCCCTATGATATTGCGGCTTCTCA | Amplification of *OG1RF_12220* gene | BamHI |
| 12220-R | CCGCTCGAGTTCCTTCTTATTTCTCGGAGT |  | XhoI |
| ID12220-F | CTAGTTCAGTCGATACACGCT | Screening recombinant plasmid of pIB166-*OG1RF_12220* |  |
| ID12220-R | AGACTGTAACATTCTCACGCA |  |  |
| q12220-F | ATAAGAACCAACAAGGAA | Detection of the RNA level of *OG1RF_12220* by RT-qPCR |  |
| q12220-R | AAGAACATCATCACTACC |  |  |
| 10126-F | CGCGGATCCACCCTCTAAAGTATTGCCAAT | Amplification of *OG1RF_10126* gene | BamHI |
| 10126-R | CCGCTCGAG TAACAGATAACACAGGGACTA |  | XhoI |
| ID10126-F | TGAATACATTCATCGTCGACT | Screening recombinant plasmid of pIB166-*OG1RF_10126* |  |
| ID10126-R | GAACTAATGGGTGCTTTAGTTG |  |  |
| q10126-F | ATCGTATCGCTGTTATTC | Detection of the RNA level of *OG1RF_10126* by RT-qPCR |  |
| q10126-R | GCTTCTTCCAATGAGTAG |  |  |
| 10665-F | CGCGGATCCTCCCTTGCCGAAAGATAGCGT | Amplification of *OG1RF_10665* gene | BamHI |
| 10665-R | TGCTCTAGAAGAGAAACAATCGAGGCATCC |  | XbaI |
| ID10665-F | TTCGGTATTCGTCCAGAAGAT | Screening recombinant plasmid of pIB166-*OG1RF_10665* |  |
| ID10665-R | ATGCGTGAGAATGTTACAGTC |  |  |
| q10665-F | GATATTCATAGCGAACAAGTG | Detection of the RNA level of *OG1RF_10665* by RT-qPCR |  |
| q10665-R | GAAGTCACGAGCATCTAC |  |  |
| 10495-F | CGCGGATCCACTAGCTTAACTAAATTAGCGA | Amplification of *OG1RF_10495* gene | BamHI |
| 10495-R | CCGCTCGAGTCTGTCTCTGTCACGGAAACA |  | XhoI |
| ID10495-F | CAACGTGTCGCAATTGCACGT | Screening recombinant plasmid of pIB166-*OG1RF_10495* |  |
| ID10495-R | GTTAATGCGCCATGACAGCCA |  |  |
| q10495-F | GTCCTTCTGGTTCTGGTA | Detection of the RNA level of *OG1RF_10495* by RT-qPCR |  |
| q10495-R | CTTGGTTGATGTCCGTATT |  |  |

**^a^**Underlined sequences represent the restriction enzyme sites of primers;
