# Supplementary material for: Radezolid Is More Effective Than Linezolid Against Planktonic Cells and Inhibits Enterococcus faecalis Biofilm Formation
Source: Front Microbiol. 2020 Feb 14;11:196. doi: 10.3389/fmicb.2020.00196 (PMC7033516; doi:10.3389/fmicb.2020.00196)
Supplement: TABLE S5 — Radezolid against 52 linezolid-nonsusceptible E. faecalis clinical isolates. [file Table_5.DOCX]

**TABLE S5︱**Radezolid against 52 linezolid-nonsusceptible *E. faecalis* clinical isolates

| **Isolates** | **MIC (mg/L)** | |  | **23S rRNA mutations** | | | | ***optrA*** |
| --- | --- | --- | --- | --- | --- | --- | --- | --- |
|  | **Radezolid** | **Linezolid** |  | **R1** | **R2** | **R3** | **R4** |  |
| 16C5 | 0.25 | 4 |  | - | - | - | - | - |
| 16C10 | 0.5 | 4 |  | - | - | - | G2621T | - |
| 16C108 | 0.5 | 4 |  | - | - | - | - | - |
| 16C110 | 0.25 | 4 |  | - | - | - | - | - |
| 16C112 | 0.5 | 4 |  | - | - | - | T2245C | + |
| 16C118 | 0.25 | 4 |  | - | - | - | T2245C | - |
| 16C129 | 0.5 | 4 |  | - | - | - | T2245C | - |
| 16C131 | 0.5 | 4 |  | - | - | - | T2245C | - |
| 16C137 | 0.5 | 4 |  | - | - | - | - | - |
| 16C143 | 0.25 | 4 |  | - | - | - | G2251T | - |
| 16C146 | 0.25 | 4 |  | - | - | - | - | - |
| 16C15 | 0.5 | 8 |  | - | - | - | - | + |
| 16C154 | 1.0 | 32 |  | - | - | - | G2251T | + |
| 16C155 | 0.5 | 4 |  | - | - | G2627A | - | - |
| 16C160 | 0.25 | 4 |  | - | - | - | G2253A | - |
| 16C164 | 0.25 | 4 |  | G2576A | - | - | - | - |
| 16C168 | 0.25 | 4 |  | - | - | C2646T | - | - |
| 16C171 | 0.5 | 4 |  | - | - | - | - | - |
| 16C18 | 0.25 | 4 |  | - | - | - | - | - |
| 16C187 | 0.5 | 4 |  | - | - | - | G2251T | - |
| 16C191 | 0.25 | 4 |  | - | - | - | - | - |
| 16C202 | 0.5 | 4 |  | - | T2249A | - | - | - |
| 16C203 | 0.5 | 4 |  | - | - | - | T2245C | - |
| 16C205 | 0.5 | 4 |  | - | - | - | G2251T | - |
| 16C237 | 0.5 | 16 |  | G2576A | - | - | T2245C | - |
| 16C25 | 0.25 | 4 |  | - | - | - | G2253A | - |
| 16C263 | 0.25 | 4 |  | - | - | - | - | - |
| 16C272 | 0.5 | 8 |  | - | - | - | - | + |
| 16C285 | 0.5 | 4 |  | - | - | - | - | - |
| 16C286 | 0.5 | 4 |  | - | - | - | - | - |
| 16C29 | 0.25 | 4 |  | - | - | - | - | - |
| 16C295 | 0.5 | 4 |  | - | - | - | - | - |
| 16C298 | 0.25 | 4 |  | - | - | - | - | - |
| 16C322 | 0.5 | 4 |  | - | - | - | - | - |
| 16C326 | 0.25 | 4 |  | - | - | - | - | - |
| 16C340 | 0.5 | 8 |  | G2576A | - | - | - | - |
| 16C348 | 0.25 | 8 |  | - | - | - | - | - |
| 16C355 | 0.25 | 4 |  | - | - | - | - | - |
| 16C360 | 0.5 | 8 |  | G2505A | - | - | - | - |
| 16C377 | 0.25 | 4 |  | - | - | - | - | - |
| 16C379 | 0.25 | 4 |  | - | - | - | - | - |
| 16C394 | 0.5 | 16 |  | G2576A | - | - | - | - |
| 16C421 | 0.25 | 4 |  | - | - | - | - | - |
| 16C44 | 0.5 | 8 |  | - | - | - | - | - |
| 16C46 | 0.5 | 8 |  | - | - | - | - | - |
| 16C55 | 0.25 | 4 |  | - | - | - | - | - |
| 16C6 | 0.5 | 4 |  | - | - | - | - | - |
| 16C69 | 0.5 | 4 |  | - | - | - | - | - |
| 16C8 | 0.25 | 4 |  | - | - | - | - | - |
| 16C85 | 0.5 | 4 |  | - | - | - | - | - |
| 16C90 | 0.25 | 4 |  | - | - | - | - | - |
| 16C95 | 0.25 | 4 |  | - | - | - | G2251T | - |

+, positive; -, not detected;
