# Supplementary material for: Radezolid Is More Effective Than Linezolid Against Planktonic Cells and Inhibits Enterococcus faecalis Biofilm Formation
Source: Front Microbiol. 2020 Feb 14;11:196. doi: 10.3389/fmicb.2020.00196 (PMC7033516; doi:10.3389/fmicb.2020.00196)
Supplement: TABLE S6 — Differential RNA levels of the genes in a high-level linezolid-resistant isolate. [file Table_6.DOCX]

**TABLE S6︱**Differential RNA levels of the genes in a high-level linezolid-resistant isolate

| **Gene_ID** | **Function/Description** | **RNA levels of OG1RF-55/OG1RF-0** | **pval** | **padj** |
| --- | --- | --- | --- | --- |
| **Up-regulated** |  |  |  |  |
| *OG1RF_RS13105* | triphosphoribosyl-dephospho-CoA synthase CitG | 18.429 | 3.31E-09 | 1.62E-07 |
| *OG1RF_RS05100* | L-malate permease | 16.234 | 6.07E-17 | 1.35E-14 |
| *OG1RF_RS13130* | citrate (pro-3S)-lyase subunit beta | 13.902 | 1E-08 | 4.31E-07 |
| *OG1RF_RS13145* | hypothetical protein | 13.873 | 0.000598 | 0.004577 |
| *OG1RF_RS13125* | citrate lyase subunit alpha | 13.807 | 1.16E-06 | 2.7E-05 |
| *OG1RF_RS13120* | citrate lyase holo-[acyl-carrier protein] synthase | 12.976 | 6.78E-07 | 1.68E-05 |
| *OG1RF_RS07250* | 30S ribosomal protein S16 | 12.772 | 2.32E-24 | 5.68E-21 |
| *OG1RF_RS04565* | class C sortase | 11.179 | 5.14E-20 | 4.2E-17 |
| *OG1RF_RS13165* | citrate transporter | 11.003 | 1.62E-09 | 8.89E-08 |
| *OG1RF_RS13150* | sodium ion-translocating decarboxylase subunit beta | 10.358 | 1.61E-05 | 0.000227 |
| *OG1RF_RS12475* | hypothetical protein | 9.710 | 5.75E-19 | 1.76E-16 |
| *OG1RF_RS11380* | multidrug ABC superfamily ATP binding cassette transporter, ABC protein | 9.046 | 7.67E-06 | 0.000121 |
| *OG1RF_RS05095* | NADP-dependent malic enzyme | 8.983 | 3.51E-20 | 4.2E-17 |
| *OG1RF_RS10645* | 50S ribosomal protein L10 | 8.825 | 1.23E-19 | 6.01E-17 |
| *OG1RF_RS00845* | 30S ribosomal protein S5 | 8.701 | 2.34E-05 | 0.00031 |
| *OG1RF_RS11405* | cold-shock protein | 8.530 | 3.9E-07 | 1.06E-05 |
| *OG1RF_RS07245* | KH domain-containing protein | 8.524 | 8.19E-18 | 2.01E-15 |
| *OG1RF_RS13110* | NADP-dependent malic enzyme | 8.210 | 3.26E-05 | 0.000407 |
| *OG1RF_RS13160* | hypothetical protein | 8.199 | 0.002576 | 0.014813 |
| *OG1RF_RS09580* | DUF47 domain-containing protein | 8.191 | 5E-07 | 1.3E-05 |
| *OG1RF_RS10640* | 50S ribosomal protein L7/L12 | 7.524 | 6.59E-09 | 3.11E-07 |
| *OG1RF_RS00830* | 30S ribosomal protein S8 | 7.276 | 0.000607 | 0.004631 |
| *OG1RF_RS00865* | adenylate kinase | 7.074 | 6.71E-05 | 0.000772 |
| *OG1RF_RS00855* | 50S ribosomal protein L15 | 7.046 | 8.84E-05 | 0.000975 |
| *OG1RF_RS00840* | 50S ribosomal protein L18 | 6.966 | 0.000423 | 0.003456 |
| *OG1RF_RS00795* | 50S ribosomal protein L16 | 6.962 | 3.71E-06 | 6.54E-05 |
| *OG1RF_RS00780* | 30S ribosomal protein S19 | 6.960 | 0.003098 | 0.017289 |
| *OG1RF_RS11965* | 30S ribosomal protein S15 | 6.931 | 9.85E-16 | 2.01E-13 |
| *OG1RF_RS00805* | 30S ribosomal protein S17 | 6.865 | 2.52E-05 | 0.000326 |
| *OG1RF_RS00725* | 30S ribosomal protein S12 | 6.857 | 1.25E-05 | 0.000186 |
| *OG1RF_RS00835* | 50S ribosomal protein L6 | 6.798 | 0.000347 | 0.003005 |
| *OG1RF_RS03730* | DUF3397 domain-containing protein | 6.696 | 0.001767 | 0.011016 |
| *OG1RF_RS05510* | holin | 6.528 | 6.02E-07 | 1.52E-05 |
| *OG1RF_RS00890* | DNA-directed RNA polymerase subunit alpha | 6.376 | 1.53E-12 | 1.87E-10 |
| *OG1RF_RS00540* | reactive intermediate/imine deaminase | 6.358 | 4.7E-07 | 1.25E-05 |
| *OG1RF_RS00635* | ABC superfamily ATP binding cassette transporter, ABC protein | 6.334 | 6.61E-06 | 0.000108 |
| *OG1RF_RS00755* | 30S ribosomal protein S10 | 6.321 | 2.15E-07 | 6.27E-06 |
| *OG1RF_RS05410* | 50S ribosomal protein L7ae | 6.242 | 2.71E-05 | 0.000348 |
| *OG1RF_RS00820* | 50S ribosomal protein L5 | 6.191 | 9.4E-05 | 0.001014 |
| *OG1RF_RS13140* | [citrate (pro-3S)-lyase] ligase | 6.128 | 6.41E-06 | 0.000105 |
| *Novel00004* |  | 6.102 | 7.59E-12 | 7.53E-10 |
| *OG1RF_RS11840* | peptide ABC transporter substrate-binding protein | 6.100 | 1.5E-12 | 1.87E-10 |
| *OG1RF_RS05475* | phage major tail protein, TP901-1 family | 6.017 | 4.42E-07 | 1.19E-05 |
| *OG1RF_RS00810* | 50S ribosomal protein L14 | 5.943 | 0.000244 | 0.002233 |
| *OG1RF_RS00850* | 50S ribosomal protein L30 | 5.865 | 0.001743 | 0.010895 |
| *OG1RF_RS08830* | glycosyltransferase family 2 protein | 5.862 | 9.13E-13 | 1.32E-10 |
| *OG1RF_RS13170* | GntR family transcriptional regulator | 5.849 | 1.56E-05 | 0.000223 |
| *OG1RF_RS09020* | carbohydrate ABC transporter permease | 5.831 | 3.16E-09 | 1.58E-07 |
| *OG1RF_RS00790* | 30S ribosomal protein S3 | 5.824 | 0.00039 | 0.003274 |
| *OG1RF_RS03640* | 50S ribosomal protein L21 | 5.809 | 3.17E-13 | 5.18E-11 |
| *OG1RF_RS00895* | 50S ribosomal protein L17 | 5.770 | 5.39E-13 | 8.25E-11 |
| *OG1RF_RS08835* | methyltransferase domain-containing protein | 5.693 | 3.53E-12 | 4.11E-10 |
| *OG1RF_RS00825* | 30S ribosomal protein S14 type Z | 5.543 | 0.002523 | 0.014578 |
| *OG1RF_RS00730* | 30S ribosomal protein S7 | 5.541 | 2.05E-05 | 0.000279 |
| *OG1RF_RS00815* | 50S ribosomal protein L24 | 5.463 | 0.000348 | 0.003005 |
| *OG1RF_RS03455* | ABC superfamily ATP binding cassette transporter, ABC protein | 5.449 | 0.000149 | 0.001486 |
| *OG1RF_RS06055* | alanine--tRNA ligase | 5.384 | 6.04E-12 | 6.43E-10 |
| *OG1RF_RS00760* | 50S ribosomal protein L3 | 5.287 | 2.68E-06 | 5.13E-05 |
| *OG1RF_RS00885* | 30S ribosomal protein S11 | 5.224 | 2.2E-08 | 8.83E-07 |
| *OG1RF_RS10880* | recombination protein RecR | 5.224 | 3.35E-05 | 0.000414 |
| *OG1RF_RS00735* | elongation factor G | 5.204 | 1.28E-05 | 0.000189 |
| *OG1RF_RS05500* | structural protein | 5.186 | 1.93E-07 | 5.84E-06 |
| *OG1RF_RS08370* | aspartate--tRNA ligase | 5.152 | 2.91E-11 | 2.64E-09 |
| *OG1RF_RS08375* | histidine--tRNA ligase | 5.070 | 4.97E-08 | 1.83E-06 |
| *OG1RF_RS02300* | 1-phosphofructokinase | 4.992 | 0.007777 | 0.034516 |
| *OG1RF_RS09575* | ChbG/HpnK family deacetylase | 4.937 | 5.09E-11 | 4.46E-09 |
| *OG1RF_RS05480* | hypothetical protein | 4.907 | 1.7E-06 | 3.63E-05 |
| *OG1RF_RS05495* | phage tail family protein | 4.877 | 3.39E-06 | 6.14E-05 |
| *OG1RF_RS11520* | PRD domain-containing protein | 4.876 | 4.87E-07 | 1.28E-05 |
| *OG1RF_RS09845* | DUF5011 domain-containing protein | 4.834 | 7.18E-08 | 2.44E-06 |
| *OG1RF_RS00740* | elongation factor Tu | 4.833 | 3.04E-07 | 8.55E-06 |
| *OG1RF_RS05505* | hypothetical protein | 4.805 | 2E-09 | 1.07E-07 |
| *OG1RF_RS00785* | 50S ribosomal protein L22 | 4.799 | 0.001006 | 0.00683 |
| *OG1RF_RS00800* | 50S ribosomal protein L29 | 4.774 | 0.00165 | 0.010439 |
| *OG1RF_RS06810* | alpha-glucosidase | 4.766 | 1.49E-07 | 4.68E-06 |
| *OG1RF_RS11510* | PTS ascorbate transporter subunit IIC | 4.762 | 0.000435 | 0.003504 |
| *OG1RF_RS05700* | 2-hydroxyglutaryl-CoA dehydratase | 4.702 | 3.79E-10 | 2.82E-08 |
| *OG1RF_RS06645* | shikimate kinase | 4.690 | 5.98E-07 | 1.52E-05 |
| *OG1RF_RS04675* | phenylalanine--tRNA ligase subunit beta | 4.682 | 2.93E-10 | 2.31E-08 |
| *OG1RF_RS06870* | DNA topoisomerase IV subunit A | 4.637 | 3.91E-10 | 2.82E-08 |
| *OG1RF_RS05490* | hypothetical protein | 4.613 | 7.28E-10 | 4.35E-08 |
| *OG1RF_RS05995* | cold-shock protein | 4.574 | 0.00414 | 0.021877 |
| *OG1RF_RS00880* | 30S ribosomal protein S13 | 4.522 | 4.09E-06 | 7.11E-05 |
| *OG1RF_RS00770* | 50S ribosomal protein L23 | 4.492 | 3.63E-05 | 0.00044 |
| *OG1RF_RS06850* | DUF1803 domain-containing protein | 4.489 | 7.62E-07 | 1.85E-05 |
| *OG1RF_RS03925* | phosphotransferase | 4.380 | 1.55E-09 | 8.81E-08 |
| *OG1RF_RS10060* | inosine-uridine preferring nucleoside hydrolase | 4.358 | 8.86E-09 | 3.95E-07 |
| *OG1RF_RS09015* | ABC transporter substrate-binding protein | 4.351 | 4.45E-10 | 2.92E-08 |
| *OG1RF_RS02620* | ABC superfamily ATP binding cassette transporter, ABC protein | 4.349 | 1.89E-06 | 3.93E-05 |
| *OG1RF_RS00045* | 30S ribosomal protein S6 | 4.348 | 6.38E-10 | 3.91E-08 |
| *OG1RF_RS10650* | 50S ribosomal protein L1 | 4.338 | 5.84E-10 | 3.67E-08 |
| *OG1RF_RS00775* | 50S ribosomal protein L2 | 4.295 | 9.02E-05 | 0.000987 |
| *OG1RF_RS03645* | ribosomal-processing cysteine protease Prp | 4.277 | 2.58E-09 | 1.32E-07 |
| *OG1RF_RS04825* | CTP synthetase | 4.272 | 1.63E-09 | 8.89E-08 |
| *OG1RF_RS11515* | PTS lactose transporter subunit IIB | 4.213 | 0.000461 | 0.003663 |
| *OG1RF_RS09000* | DNA-binding response regulator | 4.181 | 9.96E-06 | 0.000152 |
| *OG1RF_RS00870* | translation initiation factor IF-1 | 4.173 | 0.000471 | 0.003724 |
| *OG1RF_RS00510* | Crp/Fnr family transcriptional regulator | 4.128 | 0.004254 | 0.022092 |
| *OG1RF_RS02810* | leucine--tRNA ligase | 4.124 | 8.78E-09 | 3.95E-07 |
| *OG1RF_RS05485* | hypothetical protein | 4.117 | 0.012141 | 0.048684 |
| *OG1RF_RS05105* | sensor histidine kinase | 4.079 | 0.001385 | 0.009004 |
| *OG1RF_RS05300* | DUF814 domain-containing protein | 4.041 | 3.02E-05 | 0.000383 |
| *OG1RF_RS03300* | peptide ABC transporter substrate-binding protein | 4.028 | 2.02E-08 | 8.39E-07 |
| *OG1RF_RS05470* | structural protein | 3.999 | 1.58E-05 | 0.000224 |
| *OG1RF_RS00765* | 50S ribosomal protein L4 | 3.987 | 1.82E-07 | 5.57E-06 |
| *OG1RF_RS10655* | 50S ribosomal protein L11 | 3.941 | 9.23E-09 | 4.04E-07 |
| *OG1RF_RS09420* | glycine--tRNA ligase subunit alpha | 3.845 | 2.28E-06 | 4.45E-05 |
| *OG1RF_RS03765* | undecaprenyldiphospho-muramoylpentapeptide beta-N-acetylglucosaminyltransferase | 3.837 | 0.010795 | 0.044301 |
| *OG1RF_RS04670* | phenylalanine--tRNA ligase subunit alpha | 3.773 | 3.11E-06 | 5.73E-05 |
| *OG1RF_RS00050* | single-stranded DNA-binding protein | 3.760 | 3.68E-08 | 1.39E-06 |
| *OG1RF_RS05405* | DUF448 domain-containing protein | 3.697 | 0.000406 | 0.003369 |
| *OG1RF_RS09415* | glycine--tRNA ligase subunit beta | 3.688 | 1.16E-07 | 3.73E-06 |
| *OG1RF_RS08485* | 23S rRNA (adenine(2503)-C(2))-methyltransferase RlmN | 3.677 | 9.15E-07 | 2.18E-05 |
| *OG1RF_RS05415* | translation initiation factor IF-2 | 3.675 | 1.31E-05 | 0.000191 |
| *OG1RF_RS11960* | polyribonucleotide nucleotidyltransferase | 3.659 | 2.18E-08 | 8.83E-07 |
| *OG1RF_RS12005* | signal peptidase I | 3.644 | 2.84E-07 | 8.08E-06 |
| *OG1RF_RS00495* | arginine deiminase | 3.628 | 0.000648 | 0.004901 |
| *OG1RF_RS11990* | 30S ribosomal protein S4 | 3.598 | 6.12E-08 | 2.15E-06 |
| *OG1RF_RS07305* | alpha-mannosidase | 3.587 | 0.009225 | 0.039442 |
| *OG1RF_RS06690* | thymidylate synthase | 3.578 | 2.1E-06 | 4.25E-05 |
| *OG1RF_RS05400* | transcription termination/antitermination protein NusA | 3.566 | 0.005873 | 0.028166 |
| *OG1RF_RS12715* | DNA-directed RNA polymerase subunit beta' | 3.538 | 4.37E-06 | 7.48E-05 |
| *OG1RF_RS00500* | ornithine carbamoyltransferase | 3.515 | 0.000864 | 0.006081 |
| *OG1RF_RS06805* | PTS beta-glucoside transporter subunit IIBCA | 3.486 | 7.47E-08 | 2.51E-06 |
| *OG1RF_RS06855* | manganese-dependent inorganic pyrophosphatase | 3.453 | 1.74E-07 | 5.41E-06 |
| *OG1RF_RS09825* | hypothetical protein | 3.446 | 8.01E-05 | 0.0009 |
| *OG1RF_RS13115* | oxaloacetate decarboxylase | 3.422 | 3.59E-14 | 6.28E-12 |
| *OG1RF_RS06650* | prephenate dehydratase | 3.414 | 7.53E-06 | 0.00012 |
| *OG1RF_RS10065* | ECF transporter S component | 3.409 | 2.15E-06 | 4.32E-05 |
| *OG1RF_RS06230* | NAD(P)/FAD-dependent oxidoreductase | 3.379 | 1.79E-06 | 3.75E-05 |
| *OG1RF_RS00505* | carbamate kinase 1 | 3.358 | 0.006073 | 0.028875 |
| *OG1RF_RS10890* | cobalt ABC transporter permease | 3.349 | 4.8E-06 | 8.11E-05 |
| *OG1RF_RS05450* | ATP-binding protein | 3.344 | 0.000147 | 0.00148 |
| *OG1RF_RS09915* | flavocytochrome c | 3.343 | 8.22E-08 | 2.69E-06 |
| *OG1RF_RS07045* | ribonuclease HII | 3.305 | 7.55E-06 | 0.00012 |
| *OG1RF_RS06635* | prephenate dehydrogenase | 3.275 | 7.61E-05 | 0.000863 |
| *OG1RF_RS13010* | redox-regulated ATPase YchF | 3.257 | 1.09E-06 | 2.57E-05 |
| *OG1RF_RS09375* | UMP kinase | 3.225 | 1.53E-06 | 3.38E-05 |
| *OG1RF_RS09835* | hypothetical protein | 3.212 | 0.000211 | 0.001986 |
| *OG1RF_RS03770* | cell division protein DivIB | 3.205 | 0.001657 | 0.010439 |
| *OG1RF_RS12720* | DNA-directed RNA polymerase subunit beta | 3.184 | 2.29E-06 | 4.45E-05 |
| *OG1RF_RS13020* | chromosome partitioning protein ParB | 3.179 | 1.08E-05 | 0.000162 |
| *OG1RF_RS10295* | GntR family transcriptional regulator | 3.161 | 1.45E-05 | 0.000209 |
| *OG1RF_RS05945* | glycerol dehydrogenase | 3.155 | 2.09E-06 | 4.25E-05 |
| *OG1RF_RS00565* | ATP-binding protein | 3.153 | 0.00104 | 0.007006 |
| *OG1RF_RS00575* | hypothetical protein | 3.147 | 0.001614 | 0.010246 |
| *OG1RF_RS03650* | 50S ribosomal protein L27 | 3.141 | 7.01E-07 | 1.72E-05 |
| *OG1RF_RS09300* | proline--tRNA ligase | 3.140 | 2.25E-06 | 4.45E-05 |
| *OG1RF_RS12790* | FAD:protein FMN transferase | 3.134 | 9.46E-06 | 0.000146 |
| *OG1RF_RS06060* | tRNA (adenine(22)-N(1))-methyltransferase TrmK | 3.112 | 0.000128 | 0.001307 |
| *OG1RF_RS06815* | sucrose-6-phosphate hydrolase | 3.109 | 3.06E-06 | 5.67E-05 |
| *OG1RF_RS08720* | pyruvate:ferredoxin (flavodoxin) oxidoreductase | 3.108 | 1.91E-06 | 3.94E-05 |
| *OG1RF_RS00055* | 30S ribosomal protein S18 | 3.094 | 2.85E-06 | 5.42E-05 |
| *OG1RF_RS12000* | biotin transporter BioY | 3.077 | 0.004035 | 0.021491 |
| *OG1RF_RS05045* | hypothetical protein | 3.067 | 2.53E-05 | 0.000326 |
| *OG1RF_RS07500* | UTP--glucose-1-phosphate uridylyltransferase | 3.065 | 3.66E-06 | 6.5E-05 |
| *OG1RF_RS10885* | thiaminase II | 3.063 | 5.27E-06 | 8.79E-05 |
| *OG1RF_RS09025* | sugar ABC transporter permease | 3.048 | 1.33E-06 | 3.03E-05 |
| *OG1RF_RS06700* | tRNA epoxyqueuosine(34) reductase QueG | 3.039 | 9.15E-06 | 0.000142 |
| *OG1RF_RS09590* | 30S ribosomal protein S20 | 3.031 | 0.003008 | 0.016862 |
| *OG1RF_RS09730* | arginine--tRNA ligase | 3.005 | 2.17E-06 | 4.32E-05 |
| *OG1RF_RS00860* | preprotein translocase subunit SecY | 2.998 | 0.000365 | 0.003127 |
| *OG1RF_RS06320* | fructose-bisphosphatase class III | 2.971 | 0.007438 | 0.033809 |
| *OG1RF_RS06180* | endonuclease MutS2 | 2.968 | 7.06E-06 | 0.000114 |
| *OG1RF_RS08545* | AraC family transcriptional regulator | 2.965 | 2.9E-05 | 0.00037 |
| *OG1RF_RS08070* | UDP-N-acetylmuramate--L-alanine ligase | 2.963 | 3.53E-06 | 6.31E-05 |
| *OG1RF_RS13005* | DUF1129 domain-containing protein | 2.960 | 3.86E-06 | 6.76E-05 |
| *OG1RF_RS07460* | Tyrosine--tRNA ligase 2 | 2.957 | 3.3E-06 | 6.02E-05 |
| *OG1RF_RS08815* | sugar transferase | 2.942 | 8.49E-05 | 0.000945 |
| *OG1RF_RS06560* | ribosome biogenesis GTPase Der | 2.941 | 2.22E-05 | 0.000296 |
| *OG1RF_RS05465* | transcriptional regulator | 2.937 | 0.000998 | 0.006789 |
| *OG1RF_RS01550* | serine protease | 2.934 | 5.51E-06 | 9.12E-05 |
| *OG1RF_RS12815* | geranylgeranyl pyrophosphate synthase | 2.925 | 7.83E-05 | 0.000884 |
| *OG1RF_RS06860* | pyruvate formate lyase-activating protein | 2.919 | 1.59E-06 | 3.45E-05 |
| *OG1RF_RS08995* | cell wall surface anchor family antigen | 2.902 | 1.19E-05 | 0.000177 |
| *OG1RF_RS06985* | iron ABC transporter permease | 2.886 | 0.000168 | 0.001649 |
| *OG1RF_RS00925* | energy-coupling factor transporter transmembrane protein EcfT | 2.882 | 0.000229 | 0.002109 |
| *OG1RF_RS03960* | 6-aminohexanoate hydrolase | 2.876 | 1.7E-05 | 0.000236 |
| *OG1RF_RS09585* | inorganic phosphate transporter | 2.873 | 0.00275 | 0.015594 |
| *OG1RF_RS10955* | galactose-1-phosphate uridylyltransferase | 2.873 | 3.49E-05 | 0.000428 |
| *OG1RF_RS08855* | DUF2304 domain-containing protein | 2.855 | 0.004992 | 0.024959 |
| *OG1RF_RS03915* | Pyruvate phosphate dikinase | 2.844 | 3.14E-05 | 0.000395 |
| *OG1RF_RS00040* | DNA gyrase subunit A | 2.833 | 0.000922 | 0.006396 |
| *OG1RF_RS06140* | molybdenum cofactor biosynthesis protein MoaB | 2.816 | 0.00037 | 0.003151 |
| *OG1RF_RS10075* | serine hydrolase | 2.811 | 0.001781 | 0.011075 |
| *OG1RF_RS12795* | FMN-binding domain-containing protein | 2.809 | 4.09E-05 | 0.000489 |
| *OG1RF_RS02540* | hypothetical protein | 2.788 | 0.001084 | 0.007274 |
| *OG1RF_RS13015* | DUF951 domain-containing protein | 2.786 | 0.001151 | 0.007685 |
| *OG1RF_RS08860* | glycosyltransferase family 2 protein | 2.785 | 0.000335 | 0.002925 |
| *OG1RF_RS12800* | NAD(P)/FAD-dependent oxidoreductase | 2.785 | 5.26E-06 | 8.79E-05 |
| *OG1RF_RS00515* | YfcC family protein | 2.784 | 0.009346 | 0.039892 |
| *OG1RF_RS04760* | 2, 3, 4, 5-tetrahydropyridine-2, 6-dicarboxylate N-acetyltransferase | 2.770 | 0.004475 | 0.022985 |
| *OG1RF_RS09235* | asparagine--tRNA ligase | 2.759 | 1.01E-05 | 0.000153 |
| *OG1RF_RS01580* | DUF979 domain-containing protein | 2.753 | 0.000273 | 0.002424 |
| *OG1RF_RS03510* | phosphate acetyltransferase | 2.732 | 0.005959 | 0.028461 |
| *OG1RF_RS12235* | peptide deformylase | 2.708 | 0.00489 | 0.0246 |
| *OG1RF_RS12100* | cupin | 2.684 | 0.000271 | 0.002418 |
| *OG1RF_RS07425* | multidrug ABC superfamily ATP binding cassette transporter, ABC protein | 2.678 | 8.69E-05 | 0.000963 |
| *OG1RF_RS07435* | hypothetical protein | 2.643 | 0.000747 | 0.005429 |
| *OG1RF_RS12785* | 1,4-dihydroxy-2-naphthoate polyprenyltransferase | 2.624 | 9.95E-05 | 0.001069 |
| *OG1RF_RS04940* | transcription termination factor Rho | 2.618 | 0.00012 | 0.001248 |
| *OG1RF_RS07430* | multidrug ABC superfamily ATP binding cassette transporter, ABC protein | 2.595 | 0.009647 | 0.040682 |
| *OG1RF_RS07295* | phosphate ABC transporter substrate-binding protein | 2.592 | 0.000221 | 0.002063 |
| *OG1RF_RS03810* | isoleucine--tRNA ligase | 2.581 | 6.19E-05 | 0.000715 |
| *OG1RF_RS09260* | amino acid permease | 2.576 | 9.19E-05 | 0.000996 |
| *OG1RF_RS10700* | transcription termination/antitermination protein NusG | 2.566 | 0.000317 | 0.002797 |
| *OG1RF_RS00160* | copper ABC transporter permease | 2.565 | 0.000134 | 0.001367 |
| *OG1RF_RS03345* | 50S ribosomal protein L20 | 2.565 | 0.000127 | 0.001307 |
| *OG1RF_RS06990* | ABC transporter substrate-binding protein | 2.549 | 0.000204 | 0.001933 |
| *OG1RF_RS06980* | ABC transporter ATP-binding protein | 2.546 | 0.000788 | 0.005697 |
| *OG1RF_RS10980* | rhodanese-like domain-containing protein | 2.542 | 0.000426 | 0.003463 |
| *OG1RF_RS09630* | PDZ domain-containing protein | 2.541 | 0.000151 | 0.001494 |
| *OG1RF_RS03320* | ABC superfamily ATP binding cassette transporter, ABC protein | 2.537 | 0.0002 | 0.001895 |
| *OG1RF_RS09895* | threonylcarbamoyl-AMP synthase | 2.533 | 0.000819 | 0.005871 |
| *OG1RF_RS08840* | ABC superfamily ATP binding cassette transporter, ABC protein | 2.532 | 0.000827 | 0.005906 |
| *OG1RF_RS09830* | FtsW/RodA/SpoVE family cell cycle protein | 2.524 | 0.003947 | 0.021113 |
| *OG1RF_RS08550* | DNA topoisomerase III | 2.523 | 0.000416 | 0.003425 |
| *OG1RF_RS04830* | penicillin-binding protein 1A | 2.521 | 0.000216 | 0.002022 |
| *OG1RF_RS01835* | acetate--CoA ligase | 2.515 | 5.8E-05 | 0.000674 |
| *OG1RF_RS08870* | dTDP-glucose 4, 6-dehydratase | 2.506 | 7.19E-05 | 0.000819 |
| *OG1RF_RS02380* | phosphocarrier protein HPr | 2.500 | 0.000479 | 0.003773 |
| *OG1RF_RS08385* | D-aminoacyl-tRNA deacylase | 2.496 | 0.000376 | 0.003176 |
| *OG1RF_RS09380* | elongation factor Ts | 2.495 | 3.55E-05 | 0.000432 |
| *OG1RF_RS05515* | endolysin | 2.494 | 0.00192 | 0.011698 |
| *OG1RF_RS01780* | peptide MFS transporter | 2.491 | 0.000496 | 0.003893 |
| *OG1RF_RS10330* | GntP family permease | 2.482 | 0.002596 | 0.014859 |
| *OG1RF_RS06640* | 3-phosphoshikimate 1-carboxyvinyltransferase | 2.475 | 0.000392 | 0.003274 |
| *OG1RF_RS09370* | ribosome-recycling factor | 2.470 | 0.000116 | 0.001218 |
| *OG1RF_RS06605* | thioesterase | 2.466 | 0.002255 | 0.013292 |
| *OG1RF_RS08880* | glucose-1-phosphate thymidylyltransferase | 2.465 | 0.001236 | 0.008184 |
| *OG1RF_RS03750* | cell division protein FtsI | 2.462 | 0.000109 | 0.00117 |
| *OG1RF_RS12115* | chromosome segregation protein SMC | 2.451 | 0.001342 | 0.008813 |
| *OG1RF_RS02470* | 23S rRNA (uracil(1939)-C(5))-methyltransferase RlmD | 2.448 | 0.006963 | 0.032068 |
| *OG1RF_RS05530* | N-acetyltransferase | 2.447 | 0.004613 | 0.023547 |
| *OG1RF_RS11165* | acetyl-CoA carboxylase biotin carboxyl carrier protein | 2.443 | 0.000757 | 0.005485 |
| *OG1RF_RS02410* | hypothetical protein | 2.432 | 0.000333 | 0.002917 |
| *OG1RF_RS02310* | PTS fructose transporter subunit IIA | 2.423 | 0.000262 | 0.002358 |
| *OG1RF_RS11435* | valine--tRNA ligase | 2.417 | 0.000147 | 0.001478 |
| *OG1RF_RS03240* | glutamate ABC transporter permease | 2.407 | 0.000256 | 0.00232 |
| *OG1RF_RS07505* | glycerol-3-phosphate dehydrogenase (NAD(P)(+)) | 2.399 | 0.005429 | 0.026445 |
| *OG1RF_RS02160* | rRNA pseudouridine synthase | 2.392 | 0.005032 | 0.025055 |
| *OG1RF_RS00035* | DNA topoisomerase (ATP-hydrolyzing) subunit B | 2.390 | 0.01068 | 0.043902 |
| *OG1RF_RS04005* | drug:H+ antiporter-1 family protein | 2.383 | 0.00536 | 0.026369 |
| *OG1RF_RS01925* | class 1b ribonucleoside-diphosphate reductase subunit beta | 2.383 | 0.000438 | 0.00352 |
| *OG1RF_RS05615* | SprT family protein | 2.381 | 0.000462 | 0.003663 |
| *OG1RF_RS01205* | PRD domain-containing protein | 2.378 | 0.008378 | 0.036524 |
| *OG1RF_RS05370* | LTA synthase family protein | 2.375 | 0.000244 | 0.002233 |
| *OG1RF_RS08740* | GTPase HflX | 2.366 | 0.000994 | 0.006783 |
| *OG1RF_RS10515* | hypothetical protein | 2.358 | 0.000368 | 0.003144 |
| *OG1RF_RS07580* | peptide chain release factor 2 | 2.355 | 0.001355 | 0.008875 |
| *OG1RF_RS06695* | trimethoprim-resistant dihydrofolate reductase DfrE | 2.353 | 0.001843 | 0.011372 |
| *OG1RF_RS04050* | sensor histidine kinase | 2.345 | 0.001386 | 0.009004 |
| *OG1RF_RS06875* | DNA topoisomerase IV subunit B | 2.339 | 0.000638 | 0.00484 |
| *OG1RF_RS06615* | shikimate dehydrogenase | 2.336 | 0.004098 | 0.021731 |
| *OG1RF_RS04035* | 50S ribosomal protein L32 | 2.336 | 0.00018 | 0.001746 |
| *OG1RF_RS04550* | von Willebrand factor type A domain protein | 2.331 | 5.22E-07 | 1.35E-05 |
| *OG1RF_RS08820* | hypothetical protein | 2.316 | 0.004467 | 0.022985 |
| *OG1RF_RS13025* | ParA family protein | 2.303 | 0.001918 | 0.011698 |
| *OG1RF_RS12990* | serine--tRNA ligase | 2.301 | 0.000178 | 0.001732 |
| *OG1RF_RS05865* | ABC superfamily ATP binding cassette transporter, ABC protein | 2.296 | 0.003445 | 0.018968 |
| *OG1RF_RS11700* | linear amide C-N hydrolase | 2.286 | 0.005875 | 0.028166 |
| *OG1RF_RS03020* | ATP-dependent helicase | 2.275 | 0.000389 | 0.003274 |
| *OG1RF_RS09085* | glucuronyl hydrolase | 2.265 | 0.001924 | 0.011698 |
| *OG1RF_RS11945* | rod shape-determining protein MreD | 2.264 | 0.001674 | 0.010488 |
| *OG1RF_RS02225* | N-acetyltransferase | 2.260 | 0.00205 | 0.012217 |
| *OG1RF_RS09820* | glycine cleavage system protein H | 2.257 | 0.004738 | 0.024035 |
| *OG1RF_RS11690* | hypothetical protein | 2.251 | 0.012366 | 0.049344 |
| *OG1RF_RS01430* | septation ring formation regulator EzrA | 2.249 | 0.000417 | 0.003425 |
| *OG1RF_RS07095* | branched-chain phosphotransacylase | 2.248 | 0.001556 | 0.009979 |
| *OG1RF_RS06025* | bifunctional oligoribonuclease/PAP phosphatase NrnA | 2.244 | 0.000687 | 0.005099 |
| *OG1RF_RS13185* | protein translocase component YidC | 2.242 | 0.00539 | 0.026372 |
| *OG1RF_RS04960* | fructokinase | 2.238 | 0.000553 | 0.004264 |
| *OG1RF_RS03415* | methionine--tRNA ligase | 2.236 | 0.000454 | 0.003626 |
| *OG1RF_RS06030* | hypothetical protein | 2.233 | 0.000515 | 0.00403 |
| *OG1RF_RS07320* | LysR family transcriptional regulator | 2.227 | 0.001964 | 0.011912 |
| *OG1RF_RS06455* | GTPase ObgE | 2.226 | 0.000724 | 0.005311 |
| *OG1RF_RS09665* | FtsW/RodA/SpoVE family cell cycle protein | 2.223 | 0.002027 | 0.012143 |
| *OG1RF_RS00075* | adenylosuccinate synthetase | 2.221 | 0.00262 | 0.01494 |
| *OG1RF_RS07050* | ribosome biogenesis GTPase YlqF | 2.218 | 0.011848 | 0.047665 |
| *OG1RF_RS09305* | protease eep | 2.218 | 0.000841 | 0.005975 |
| *OG1RF_RS06865* | formate C-acetyltransferase | 2.217 | 0.004198 | 0.022072 |
| *OG1RF_RS01215* | 6-phospho-beta-glucosidase | 2.214 | 0.001976 | 0.011925 |
| *OG1RF_RS01265* | enoyl-[acyl-carrier-protein] reductase FabI | 2.214 | 0.000445 | 0.003563 |
| *OG1RF_RS03340* | 50S ribosomal protein L35 | 2.211 | 0.001326 | 0.008731 |
| *OG1RF_RS10475* | tryptophan--tRNA ligase | 2.210 | 0.00082 | 0.005871 |
| *OG1RF_RS06600* | CCA-adding enzyme | 2.203 | 0.003874 | 0.020862 |
| *OG1RF_RS09890* | serine hydroxymethyltransferase | 2.193 | 0.000676 | 0.005044 |
| *OG1RF_RS05695* | TetR/AcrR family transcriptional regulator | 2.170 | 0.002516 | 0.014571 |
| *OG1RF_RS05625* | pyridoxal phosphate-dependent aminotransferase | 2.169 | 0.000873 | 0.006118 |
| *OG1RF_RS12685* | 30S ribosomal protein S9 | 2.155 | 0.000836 | 0.005951 |
| *OG1RF_RS11045* | penicillin-binding protein 2 | 2.152 | 0.00192 | 0.011698 |
| *OG1RF_RS07510* | prolipoprotein diacylglyceryl transferase | 2.149 | 0.00765 | 0.034031 |
| *OG1RF_RS08865* | dTDP-4-dehydrorhamnose reductase | 2.148 | 0.002163 | 0.012829 |
| *OG1RF_RS11385* | ABC superfamily ATP binding cassette transporter, ABC/membrane protein | 2.145 | 0.002593 | 0.014859 |
| *OG1RF_RS11160* | beta-hydroxyacyl-ACP dehydratase | 2.140 | 0.00355 | 0.019366 |
| *OG1RF_RS04555* | cell wall surface anchor family protein | 2.127 | 1.51E-09 | 8.8E-08 |
| *OG1RF_RS09355* | cysteine desulfurase | 2.113 | 0.003303 | 0.018308 |
| *OG1RF_RS12105* | signal recognition particle-docking protein FtsY | 2.103 | 0.001668 | 0.010477 |
| *OG1RF_RS12375* | DUF4115 domain-containing protein | 2.103 | 0.001223 | 0.008119 |
| *OG1RF_RS02625* | glutamine ABC transporter substrate-binding protein GlnH | 2.098 | 0.003344 | 0.018459 |
| *OG1RF_RS01585* | pyroglutamyl-peptidase I | 2.096 | 0.001041 | 0.007006 |
| *OG1RF_RS06670* | hypothetical protein | 2.092 | 0.009418 | 0.04012 |
| *OG1RF_RS00965* | hypothetical protein | 2.075 | 0.001593 | 0.010135 |
| *OG1RF_RS05950* | PTS-dependent dihydroxyacetone kinase phosphotransferase subunit DhaM | 2.074 | 0.004225 | 0.022077 |
| *OG1RF_RS06285* | V-type ATP synthase subunit A | 2.074 | 0.003245 | 0.018028 |
| *OG1RF_RS09430* | GTPase Era | 2.071 | 0.00154 | 0.009903 |
| *OG1RF_RS02155* | polysaccharide biosynthesis protein | 2.070 | 0.003112 | 0.017331 |
| *OG1RF_RS11145* | acetyl-CoA carboxylase carboxyl transferase subunit alpha | 2.068 | 0.003557 | 0.019366 |
| *OG1RF_RS04865* | endonuclease III | 2.056 | 0.004733 | 0.024035 |
| *OG1RF_RS05010* | TIGR01906 family membrane protein | 2.056 | 0.006441 | 0.030116 |
| *OG1RF_RS09265* | PolC-type DNA polymerase III | 2.052 | 0.003891 | 0.020908 |
| *OG1RF_RS06235* | NADP-specific glutamate dehydrogenase | 2.045 | 0.001015 | 0.006868 |
| *OG1RF_RS11050* | threonine--tRNA ligase | 2.040 | 0.002782 | 0.015706 |
| *OG1RF_RS09500* | phospho-sugar mutase | 2.040 | 0.002225 | 0.013169 |
| *OG1RF_RS05050* | MBL fold metallo-hydrolase | 2.033 | 0.005342 | 0.026336 |
| *OG1RF_RS12690* | 50S ribosomal protein L13 | 2.032 | 0.004831 | 0.024354 |
| *OG1RF_RS10825* | anaerobic ribonucleoside-triphosphate reductase activating protein | 2.024 | 0.00197 | 0.011916 |
| *OG1RF_RS13085* | tRNA uridine-5-carboxymethylaminomethyl(34) synthesis enzyme MnmG | 2.021 | 0.009432 | 0.04012 |
| *OG1RF_RS01540* | hypothetical protein | 2.016 | 0.009538 | 0.040361 |
| *OG1RF_RS07465* | penicillin-binding protein | 2.015 | 0.002965 | 0.016663 |
| *OG1RF_RS05375* | class I SAM-dependent rRNA methyltransferase | 2.012 | 0.007619 | 0.034031 |
| *OG1RF_RS02635* | excinuclease ABC subunit UvrA | 2.007 | 0.004309 | 0.02232 |
| *OG1RF_RS11150* | acetyl-CoA carboxylase carboxyltransferase subunit beta | 2.005 | 0.003932 | 0.021078 |
| *OG1RF_RS03325* | ABC superfamily ATP binding cassette transporter, ABC protein | 2.004 | 0.004503 | 0.023081 |
|  |  |  |  |  |
| **Down-regulated** |  |  |  |  |
| *OG1RF_RS00285* | multifunctional 2', 3'-cyclic-nucleotide 2'-phosphodiesterase/5'-nucleotidase/3'-nucleotidase | 0.499 | 0.001272 | 0.008398 |
| *OG1RF_RS08275* | SIS domain-containing protein | 0.496 | 0.00673 | 0.031228 |
| *OG1RF_RS04890* | PTS system cellobiose-specific IIC component | 0.494 | 0.008066 | 0.035635 |
| *OG1RF_RS08500* | GntR family transcriptional regulator | 0.494 | 0.003993 | 0.021313 |
| *OG1RF_RS10395* | diacylglycerol kinase | 0.493 | 0.012333 | 0.04932 |
| *OG1RF_RS03035* | alanine racemase | 0.489 | 0.01018 | 0.042636 |
| *OG1RF_RS05280* | oxidoreductase | 0.489 | 0.004152 | 0.021877 |
| *OG1RF_RS11465* | NCS2 family permease | 0.488 | 0.006581 | 0.030713 |
| *OG1RF_RS08715* | TIGR00159 family protein | 0.486 | 0.00422 | 0.022077 |
| *OG1RF_RS12755* | phosphatase PAP2 family protein | 0.485 | 0.005404 | 0.026372 |
| *OG1RF_RS11915* | hypothetical protein | 0.485 | 0.006377 | 0.029872 |
| *OG1RF_RS03215* | hypothetical protein | 0.484 | 0.000994 | 0.006783 |
| *OG1RF_RS07260* | NAD(P)H-dependent oxidoreductase | 0.482 | 0.00153 | 0.009861 |
| *OG1RF_RS01920* | TPM domain-containing protein | 0.481 | 0.001796 | 0.011141 |
| *OG1RF_RS03200* | hypothetical protein | 0.478 | 0.005162 | 0.02555 |
| *OG1RF_RS00345* | KR domain-containing protein | 0.476 | 0.000625 | 0.004752 |
| *OG1RF_RS09685* | inositol monophosphatase family protein | 0.475 | 0.00986 | 0.041508 |
| *OG1RF_RS12570* | murein hydrolase regulator LrgA | 0.475 | 0.010472 | 0.043401 |
| *OG1RF_RS09215* | N-acetylmuramoyl-L-alanine amidase | 0.475 | 0.011809 | 0.047584 |
| *OG1RF_RS13050* | fructose-bisphosphate aldolase | 0.473 | 0.000699 | 0.005171 |
| *OG1RF_RS10465* | adapter protein MecA | 0.472 | 0.004955 | 0.024879 |
| *OG1RF_RS12580* | sensor protein LytS | 0.469 | 0.003553 | 0.019366 |
| *OG1RF_RS05265* | DUF5067 domain-containing protein | 0.468 | 0.005662 | 0.027414 |
| *OG1RF_RS05230* | hypothetical protein | 0.464 | 0.000882 | 0.006137 |
| *OG1RF_RS01050* | redox-regulated molecular chaperone Hsp33 | 0.462 | 0.0075 | 0.033875 |
| *OG1RF_RS08265* | DUF2200 domain-containing protein | 0.461 | 0.00759 | 0.033994 |
| *OG1RF_RS00420* | peptidase | 0.460 | 0.006927 | 0.03196 |
| *OG1RF_RS07360* | dihydroorotase | 0.459 | 0.002622 | 0.01494 |
| *OG1RF_RS08430* | universal stress protein | 0.458 | 0.000538 | 0.004169 |
| *OG1RF_RS09400* | acylphosphatase | 0.457 | 0.002267 | 0.01332 |
| *OG1RF_RS00990* | aldehyde dehydrogenase | 0.455 | 0.000653 | 0.00492 |
| *OG1RF_RS09230* | gfo/Idh/MocA family oxidoreductase | 0.454 | 0.001456 | 0.009414 |
| *OG1RF_RS11420* | bifunctional folylpolyglutamate synthase/dihydrofolate synthase | 0.454 | 0.001834 | 0.011343 |
| *OG1RF_RS10270* | co-chaperone GroES | 0.447 | 0.005393 | 0.026372 |
| *OG1RF_RS12500* | succinyl-diaminopimelate desuccinylase | 0.447 | 0.00518 | 0.025587 |
| *OG1RF_RS08280* | PTS mannose transporter subunit IID | 0.445 | 0.003345 | 0.018459 |
| *OG1RF_RS12295* | PTS mannose/fructose/sorbose transporter subunit IIB | 0.445 | 0.002562 | 0.014767 |
| *OG1RF_RS00600* | adenosine deaminase | 0.443 | 0.006081 | 0.028875 |
| *OG1RF_RS12765* | WxL domain-containing protein | 0.439 | 0.00916 | 0.039404 |
| *OG1RF_RS07800* | glycosyltransferase family 8 protein | 0.437 | 0.000271 | 0.002418 |
| *OG1RF_RS01695* | IclR family transcriptional regulator | 0.435 | 0.004628 | 0.023572 |
| *OG1RF_RS05670* | flotillin family protein | 0.433 | 0.000115 | 0.001214 |
| *OG1RF_RS08270* | glucosamine--fructose-6-phosphate aminotransferase | 0.426 | 0.000563 | 0.004323 |
| *OG1RF_RS11500* | LacI family transcriptional regulator | 0.424 | 0.007641 | 0.034031 |
| *OG1RF_RS10920* | transcriptional regulator | 0.423 | 0.010497 | 0.043401 |
| *OG1RF_RS04640* | iron-sulfur cluster-binding protein | 0.422 | 0.000287 | 0.002543 |
| *OG1RF_RS07760* | PTS fructose transporter subunit IID | 0.420 | 0.000677 | 0.005044 |
| *OG1RF_RS05115* | NADH peroxidase | 0.419 | 0.000135 | 0.001373 |
| *OG1RF_RS02050* | transcriptional regulator | 0.418 | 0.00249 | 0.014456 |
| *OG1RF_RS06725* | N-acetylmuramoyl-L-alanine amidase | 0.414 | 0.000539 | 0.004169 |
| *OG1RF_RS13290* | acyl-CoA synthetase FdrA | 0.411 | 4.86E-05 | 0.000575 |
| *OG1RF_RS11920* | class A sortase | 0.411 | 0.006198 | 0.029316 |
| *OG1RF_RS07370* | uracil transporter | 0.411 | 0.009215 | 0.039442 |
| *OG1RF_RS07070* | 2-oxo acid dehydrogenase subunit E2 | 0.409 | 0.004518 | 0.023108 |
| *OG1RF_RS04810* | lipoate--protein ligase family protein | 0.406 | 0.000955 | 0.006595 |
| *OG1RF_RS01980* | Tyrosine--tRNA ligase 1 | 0.406 | 0.000856 | 0.006044 |
| *OG1RF_RS06330* | alpha/beta hydrolase | 0.404 | 0.009483 | 0.040195 |
| *OG1RF_RS07705* | membrane protein | 0.404 | 0.000115 | 0.001214 |
| *OG1RF_RS07790* | tagatose-6-phosphate ketose | 0.403 | 0.00033 | 0.002901 |
| *OG1RF_RS02085* | nitroreductase | 0.402 | 0.000363 | 0.003123 |
| *OG1RF_RS11785* | dicarboxylate/amino acid:cation symporter | 0.399 | 0.000116 | 0.001215 |
| *OG1RF_RS03490* | N-acetyltransferase | 0.398 | 0.001426 | 0.00924 |
| *OG1RF_RS05245* | endonuclease | 0.397 | 0.002323 | 0.013581 |
| *OG1RF_RS10540* | tRNA1(Val) (adenine(37)-N6)-methyltransferase | 0.396 | 0.004971 | 0.024907 |
| *OG1RF_RS01025* | hypothetical protein | 0.394 | 0.000956 | 0.006595 |
| *OG1RF_RS03840* | ATP-dependent helicase | 0.392 | 6.82E-05 | 0.000781 |
| *OG1RF_RS06735* | transcriptional repressor | 0.392 | 0.000851 | 0.006026 |
| *OG1RF_RS01610* | PTS system protein | 0.391 | 0.000372 | 0.003151 |
| *OG1RF_RS01360* | hypothetical protein | 0.387 | 0.000173 | 0.001693 |
| *OG1RF_RS00590* | esterase | 0.387 | 0.00015 | 0.001492 |
| *OG1RF_RS07820* | LysR family transcriptional regulator | 0.386 | 0.001569 | 0.010011 |
| *OG1RF_RS05520* | tRNA pseudouridine(55) synthase TruB | 0.384 | 0.006757 | 0.031296 |
| *OG1RF_RS01520* | dicarboxylate/amino acid:cation symporter | 0.384 | 0.002435 | 0.014171 |
| *OG1RF_RS08535* | cytochrome d ubiquinol oxidase subunit II | 0.379 | 2.15E-05 | 0.000289 |
| *OG1RF_RS04485* | DUF4111 domain-containing protein | 0.378 | 0.000718 | 0.005285 |
| *OG1RF_RS12565* | antiholin | 0.374 | 3.28E-05 | 0.000408 |
| *OG1RF_RS06465* | PTS sugar transporter subunit IIC | 0.371 | 2.23E-05 | 0.000296 |
| *OG1RF_RS07590* | ribosomal subunit interface protein | 0.371 | 3.65E-05 | 0.00044 |
| *OG1RF_RS00520* | type 1 glutamine amidotransferase domain-containing protein | 0.368 | 0.001131 | 0.007568 |
| *OG1RF_RS11725* | acyl-CoA synthetase FdrA | 0.368 | 0.007547 | 0.033932 |
| *OG1RF_RS02585* | legume lectins beta domain protein | 0.365 | 0.007654 | 0.034031 |
| *OG1RF_RS11255* | hypothetical protein | 0.364 | 0.008087 | 0.035635 |
| *OG1RF_RS04515* | hypothetical protein | 0.364 | 0.000343 | 0.002976 |
| *OG1RF_RS04480* | N-acetyltransferase | 0.362 | 0.000659 | 0.004956 |
| *OG1RF_RS01535* | N-acyl-D-amino-acid deacylase | 0.361 | 0.000184 | 0.001771 |
| *OG1RF_RS02560* | DUF1003 domain-containing protein | 0.358 | 0.000433 | 0.003504 |
| *OG1RF_RS06960* | propanediol utilization protein PduU | 0.358 | 0.009195 | 0.039442 |
| *OG1RF_RS02665* | thioredoxin | 0.358 | 0.010459 | 0.043401 |
| *OG1RF_RS05110* | response regulator | 0.357 | 7.92E-08 | 2.62E-06 |
| *OG1RF_RS04520* | universal stress protein | 0.356 | 3.05E-05 | 0.000385 |
| *OG1RF_RS09875* | hypothetical protein | 0.356 | 0.000875 | 0.006118 |
| *OG1RF_RS08290* | PTS mannose/fructose/sorbose transporter subunit IIB | 0.355 | 0.001922 | 0.011698 |
| *OG1RF_RS13690* | hypothetical protein | 0.354 | 0.000427 | 0.003463 |
| *OG1RF_RS10305* | glycine/betaine ABC transporter permease | 0.353 | 0.00746 | 0.033848 |
| *OG1RF_RS11095* | hypothetical protein | 0.353 | 0.010642 | 0.043821 |
| *OG1RF_RS07365* | aspartate carbamoyltransferase | 0.350 | 0.003097 | 0.017289 |
| *OG1RF_RS06480* | DUF1093 domain-containing protein | 0.349 | 0.000263 | 0.002359 |
| *OG1RF_RS04645* | lactate utilization protein C | 0.349 | 1.71E-05 | 0.000236 |
| *OG1RF_RS01375* | flavin reductase family protein | 0.348 | 0.00444 | 0.022901 |
| *OG1RF_RS07775* | beta-galactosidase | 0.347 | 0.000189 | 0.001801 |
| *OG1RF_RS11670* | allantoinase AllB | 0.344 | 0.003596 | 0.019492 |
| *OG1RF_RS03195* | hypothetical protein | 0.344 | 0.002083 | 0.012387 |
| *OG1RF_RS00360* | Asp23/Gls24 family envelope stress response protein | 0.343 | 4.05E-05 | 0.000486 |
| *OG1RF_RS03855* | PTS sugar transporter subunit IIC | 0.338 | 8.19E-06 | 0.000129 |
| *OG1RF_RS03305* | DUF3899 domain-containing protein | 0.335 | 0.008243 | 0.036102 |
| *OG1RF_RS03150* | copper-translocating P-type ATPase | 0.335 | 4.48E-06 | 7.62E-05 |
| *OG1RF_RS06225* | ABC-F type ribosomal protection protein | 0.333 | 0.000877 | 0.006118 |
| *OG1RF_RS06965* | alcohol dehydrogenase | 0.332 | 0.012463 | 0.049465 |
| *OG1RF_RS07140* | permease | 0.329 | 0.004256 | 0.022092 |
| *OG1RF_RS01315* | 6-phospho-beta-glucosidase | 0.329 | 3.01E-06 | 5.63E-05 |
| *OG1RF_RS01525* | ureidoglycolate dehydrogenase | 0.327 | 2.43E-05 | 0.00032 |
| *OG1RF_RS13425* | RNase P RNA component class B | 0.326 | 1.55E-06 | 3.39E-05 |
| *OG1RF_RS03210* | glycosyltransferase family 2 protein | 0.325 | 5.03E-05 | 0.000593 |
| *OG1RF_RS03040* | type II toxin-antitoxin system PemK/MazF family toxin | 0.324 | 0.00735 | 0.033535 |
| *OG1RF_RS03220* | hypothetical protein | 0.324 | 0.000127 | 0.001307 |
| *OG1RF_RS04895* | glycine/betaine/sarcosine/D-proline reductase family selenoprotein B | 0.320 | 1.3E-05 | 0.00019 |
| *OG1RF_RS03120* | glyoxalase | 0.317 | 0.000127 | 0.001307 |
| *OG1RF_RS12560* | cysteine hydrolase | 0.315 | 0.000745 | 0.005429 |
| *OG1RF_RS12305* | PTS sugar transporter subunit IIC | 0.314 | 1.48E-06 | 3.3E-05 |
| *OG1RF_RS02020* | L-lactate dehydrogenase | 0.312 | 0.012425 | 0.049442 |
| *OG1RF_RS01380* | ring-cleaving dioxygenase | 0.312 | 0.000224 | 0.002078 |
| *OG1RF_RS08475* | secretion system protein E | 0.311 | 0.007188 | 0.032916 |
| *OG1RF_RS10300* | glycine betaine/L-proline ABC transporter ATP-binding protein | 0.310 | 0.004079 | 0.021675 |
| *OG1RF_RS07610* | PTS sugar transporter subunit IIB | 0.308 | 0.009555 | 0.040363 |
| *OG1RF_RS02090* | glyoxalase | 0.307 | 1.7E-06 | 3.63E-05 |
| *OG1RF_RS13045* | oleate hydratase | 0.305 | 1.41E-07 | 4.49E-06 |
| *OG1RF_RS12485* | Rrf2 family transcriptional regulator | 0.305 | 0.000405 | 0.003369 |
| *OG1RF_RS08955* | hypothetical protein | 0.304 | 0.002008 | 0.012055 |
| *OG1RF_RS06370* | transcription antiterminator BglG | 0.303 | 0.002257 | 0.013292 |
| *OG1RF_RS06795* | deoxyribodipyrimidine photo-lyase | 0.303 | 8.8E-06 | 0.000137 |
| *OG1RF_RS11620* | RND transporter | 0.300 | 0.009453 | 0.040138 |
| *OG1RF_RS07130* | carboxylesterase | 0.299 | 5.18E-05 | 0.000604 |
| *OG1RF_RS00415* | hypothetical protein | 0.298 | 0.010505 | 0.043401 |
| *OG1RF_RS04525* | hypothetical protein | 0.298 | 9E-05 | 0.000987 |
| *OG1RF_RS00120* | membrane protein | 0.296 | 1.77E-06 | 3.74E-05 |
| *OG1RF_RS03385* | VOC family protein | 0.295 | 1.31E-06 | 3E-05 |
| *OG1RF_RS03870* | DUF3284 domain-containing protein | 0.294 | 2.42E-06 | 4.67E-05 |
| *OG1RF_RS00365* | Asp23/Gls24 family envelope stress response protein | 0.293 | 5.01E-08 | 1.83E-06 |
| *OG1RF_RS02360* | DUF1827 domain-containing protein | 0.293 | 0.000213 | 0.002001 |
| *OG1RF_RS13415* | transfer-messenger RNA | 0.293 | 1.98E-07 | 5.85E-06 |
| *OG1RF_RS00995* | L-lactate dehydrogenase | 0.292 | 5.29E-08 | 1.91E-06 |
| *OG1RF_RS01790* | peptidase M23 | 0.291 | 3.79E-07 | 1.04E-05 |
| *OG1RF_RS00355* | DUF2273 domain-containing protein | 0.288 | 0.000976 | 0.006702 |
| *OG1RF_RS09175* | chaperone protein ClpB | 0.287 | 2.44E-08 | 9.63E-07 |
| *OG1RF_RS01510* | MFS transporter | 0.286 | 2.21E-07 | 6.37E-06 |
| *OG1RF_RS11925* | hypothetical protein | 0.284 | 0.001372 | 0.008962 |
| *OG1RF_RS05215* | metallophosphoesterase | 0.284 | 0.001993 | 0.011995 |
| *OG1RF_RS00350* | alkaline shock response membrane anchor protein AmaP | 0.283 | 1.89E-08 | 7.97E-07 |
| *OG1RF_RS00320* | N-acetylmannosamine-6-phosphate 2-epimerase | 0.279 | 4.36E-06 | 7.48E-05 |
| *OG1RF_RS11780* | DinB family protein | 0.278 | 3.45E-08 | 1.32E-06 |
| *OG1RF_RS01890* | superoxide dismutase [Fe] | 0.277 | 0.000253 | 0.002301 |
| *OG1RF_RS03030* | holo-ACP synthase | 0.276 | 0.000977 | 0.006702 |
| *OG1RF_RS01505* | DUF2877 domain-containing protein | 0.276 | 1.36E-05 | 0.000198 |
| *OG1RF_RS13065* | PTS sorbitol transporter subunit IIC | 0.271 | 5.13E-05 | 0.000601 |
| *OG1RF_RS07765* | PTS sugar transporter subunit IIC | 0.270 | 8.73E-07 | 2.1E-05 |
| *OG1RF_RS10315* | diacylglycerol kinase | 0.270 | 0.000188 | 0.001798 |
| *OG1RF_RS07755* | PTS fructose transporter subunit IIA | 0.270 | 2.03E-05 | 0.000278 |
| *OG1RF_RS10250* | LysM peptidoglycan-binding domain-containing protein | 0.270 | 0.002043 | 0.012208 |
| *OG1RF_RS12675* | hypothetical protein | 0.269 | 0.000183 | 0.001768 |
| *OG1RF_RS10310* | DUF697 domain-containing protein | 0.269 | 0.000677 | 0.005044 |
| *OG1RF_RS04375* | hypothetical protein | 0.268 | 0.002765 | 0.015642 |
| *OG1RF_RS03205* | hypothetical protein | 0.268 | 7.06E-08 | 2.44E-06 |
| *OG1RF_RS04510* | hypothetical protein | 0.267 | 0.007386 | 0.033635 |
| *OG1RF_RS07490* | YtxH domain-containing protein | 0.267 | 0.000142 | 0.001442 |
| *OG1RF_RS07770* | PTS system mannose/fructose/N-acetylgalactosamine-transporter subunit IIB | 0.267 | 0.007145 | 0.032782 |
| *OG1RF_RS04475* | hypothetical protein | 0.266 | 0.000518 | 0.004039 |
| *OG1RF_RS03225* | hypothetical protein | 0.262 | 2.45E-05 | 0.00032 |
| *OG1RF_RS06845* | DUF2179 domain-containing protein | 0.256 | 0.000408 | 0.00338 |
| *OG1RF_RS03655* | phosphate-starvation-inducible protein PsiE | 0.256 | 0.010267 | 0.042777 |
| *OG1RF_RS08520* | polyprenyl synthetase family protein | 0.256 | 1.22E-06 | 2.82E-05 |
| *OG1RF_RS02895* | GTP cyclohydrolase | 0.255 | 0.007556 | 0.033932 |
| *OG1RF_RS11030* | hypothetical protein | 0.254 | 0.003469 | 0.019014 |
| *OG1RF_RS05290* | iron-sulfur cluster biosynthesis family protein | 0.253 | 3.7E-07 | 1.03E-05 |
| *OG1RF_RS02900* | 50S ribosomal protein L25 | 0.253 | 0.001653 | 0.010439 |
| *OG1RF_RS04535* | thioredoxin | 0.253 | 0.001191 | 0.007926 |
| *OG1RF_RS02680* | DUF1622 domain-containing protein | 0.248 | 0.000229 | 0.002109 |
| *OG1RF_RS06730* | cysteine synthase A | 0.246 | 8.22E-09 | 3.8E-07 |
| *Novel00003* |  | 0.246 | 0.000151 | 0.001494 |
| *OG1RF_RS01530* | hypothetical protein | 0.241 | 3.46E-06 | 6.24E-05 |
| *OG1RF_RS08555* | DUF1033 domain-containing protein | 0.240 | 4.28E-05 | 0.00051 |
| *OG1RF_RS04435* | sugar O-acetyltransferase | 0.240 | 9.12E-05 | 0.000993 |
| *OG1RF_RS05340* | hypothetical protein | 0.239 | 0.004147 | 0.021877 |
| *OG1RF_RS10240* | nicotinate phosphoribosyltransferase | 0.237 | 0.000114 | 0.001214 |
| *OG1RF_RS07125* | ring-cleaving dioxygenase | 0.236 | 1.35E-06 | 3.04E-05 |
| *OG1RF_RS10590* | hypothetical protein | 0.234 | 2.12E-05 | 0.000287 |
| *OG1RF_RS07780* | tagatose-6-phosphate kinase | 0.234 | 8.16E-05 | 0.000913 |
| *OG1RF_RS01840* | organic hydroperoxide resistance protein | 0.234 | 0.000257 | 0.002326 |
| *OG1RF_RS06415* | lysozyme family protein | 0.232 | 0.001562 | 0.009991 |
| *OG1RF_RS05710* | WXG100 family type VII secretion target | 0.231 | 1.55E-05 | 0.000222 |
| *OG1RF_RS07785* | tagatose-bisphosphate aldolase | 0.229 | 2.95E-06 | 5.55E-05 |
| *OG1RF_RS10965* | DUF3042 domain-containing protein | 0.227 | 0.005387 | 0.026372 |
| *OG1RF_RS11640* | metal-sensitive transcriptional regulator | 0.226 | 6.9E-06 | 0.000112 |
| *OG1RF_RS04965* | YtxH domain-containing protein | 0.226 | 2.41E-10 | 1.97E-08 |
| *OG1RF_RS04915* | YajQ family cyclic di-GMP-binding protein | 0.226 | 4.11E-09 | 1.97E-07 |
| *OG1RF_RS03235* | ABC superfamily ATP binding cassette transporter, ABC protein | 0.222 | 3.42E-05 | 0.000421 |
| *OG1RF_RS07055* | 2-dehydropantoate 2-reductase | 0.219 | 0.007508 | 0.033875 |
| *OG1RF_RS12095* | glyoxalase | 0.214 | 4.16E-10 | 2.91E-08 |
| *OG1RF_RS03835* | gfo/Idh/MocA family oxidoreductase | 0.212 | 4.35E-10 | 2.92E-08 |
| *OG1RF_RS01300* | hypothetical protein | 0.209 | 0.004253 | 0.022092 |
| *OG1RF_RS04490* | N-acetyltransferase | 0.209 | 1.7E-05 | 0.000236 |
| *OG1RF_RS01330* | dihydrofolate reductase | 0.203 | 0.00229 | 0.013424 |
| *OG1RF_RS06450* | type I glyceraldehyde-3-phosphate dehydrogenase | 0.203 | 5.31E-12 | 5.91E-10 |
| *OG1RF_RS02845* | GlsB/YeaQ/YmgE family stress response membrane protein | 0.201 | 8.41E-11 | 7.11E-09 |
| *OG1RF_RS11320* | ABC superfamily ATP binding cassette transporter, ABC protein | 0.200 | 6.14E-08 | 2.15E-06 |
| *OG1RF_RS00370* | GlsB/YeaQ/YmgE family stress response membrane protein | 0.200 | 1.41E-11 | 1.33E-09 |
| *Novel00002* |  | 0.197 | 9.55E-06 | 0.000146 |
| *OG1RF_RS05870* | MarR family transcriptional regulator | 0.189 | 1.95E-07 | 5.84E-06 |
| *OG1RF_RS10460* | competence protein ComA | 0.187 | 2.55E-08 | 9.91E-07 |
| *OG1RF_RS12745* | chitin-binding protein | 0.170 | 6.56E-07 | 1.64E-05 |
| *OG1RF_RS02390* | DUF3324 domain-containing protein | 0.169 | 0.005023 | 0.025055 |
| *OG1RF_RS13510* | 6S RNA | 0.169 | 3.66E-10 | 2.81E-08 |
| *OG1RF_RS01395* | chitinase | 0.149 | 0.000817 | 0.005871 |
| *OG1RF_RS04590* | DUF898 domain-containing protein | 0.145 | 0.000166 | 0.001629 |
| *OG1RF_RS03900* | hypothetical protein | 0.138 | 2.25E-09 | 1.17E-07 |
| *OG1RF_RS06000* | hypothetical protein | 0.118 | 1.14E-19 | 6.01E-17 |
| *OG1RF_RS06755* | N-acetyltransferase | 0.117 | 1.47E-12 | 1.87E-10 |
| *OG1RF_RS01400* | chitin-binding protein | 0.102 | 0.000705 | 0.005203 |
| *OG1RF_RS02005* | DUF1307 domain-containing protein | 0.101 | 3.39E-18 | 9.22E-16 |
| *sRNA00010* |  | 0.100 | 0.000732 | 0.005353 |
| *OG1RF_RS02135* | hypothetical protein | 0.095 | 2.46E-05 | 0.00032 |
| *OG1RF_RS04575* | DUF378 domain-containing protein | 0.094 | 0.000418 | 0.003425 |
| *OG1RF_RS07735* | hypothetical protein | 0.089 | 4.53E-10 | 2.92E-08 |
| *OG1RF_RS01625* | hypothetical protein | 0.084 | 0.00237 | 0.013822 |
| *OG1RF_RS06825* | hypothetical protein | 0.081 | 4.12E-19 | 1.44E-16 |
| *OG1RF_RS04440* | hypothetical protein | 0.081 | 1.65E-05 | 0.000231 |
| *OG1RF_RS04430* | VOC family protein | 0.076 | 7.68E-12 | 7.53E-10 |
| *OG1RF_RS02395* | WxL domain-containing protein | 0.075 | 0.000525 | 0.004085 |
| *sRNA00011* |  | 0.074 | 8.74E-15 | 1.65E-12 |

**Note:** The *E. faecalis* OG1RF wildtype isolate (OG1RF-0, linezolid and radezolid sensitive) and the linezolid high-level resistance isolate (OG1RF-55, linezolid MIC: 256 mg/L; radezolid MIC: 8 mg/L) were inoculated and grown to the logarithmic phase (4 h), total RNA was isolated and sequenced by Illumina HiSeq 2500 sequencer. Data are the means of the results from three independent experiments.
