# Supplementary material for: Radezolid Is More Effective Than Linezolid Against Planktonic Cells and Inhibits Enterococcus faecalis Biofilm Formation
Source: Front Microbiol. 2020 Feb 14;11:196. doi: 10.3389/fmicb.2020.00196 (PMC7033516; doi:10.3389/fmicb.2020.00196)
Supplement: TABLE S7 — Radezolid and linezolid minimum inhibitory concentrations (MICs)in 13 biofilm positive E. faecalis clinical isolates. [file Table_7.DOCX]

**TABLE S7︱**Radezolid and linezolid minimum inhibitory concentrations (MICs) in 13 biofilm positive *E. faecalis* clinical isolates

| **Isolates** | **MIC (mg/L)** | |
| --- | --- | --- |
|  | **Linezolid** | **Radezolid** |
| 16C1 | 2 | 0.5 |
| 16C35 | 2 | 0.5 |
| 16C51 | 1 | 0.125 |
| 16C102 | 2 | 0.25 |
| 16C106 | 2 | 0.25 |
| 16C124 | 1 | 0.125 |
| 16C138 | 2 | 0.5 |
| 16C152 | 2 | 0.25 |
| 16C166 | 2 | 0.5 |
| 16C201 | 2 | 0.25 |
| 16C289 | 1 | 0.25 |
| 16C350 | 2 | 0.25 |
| 16C353 | 2 | 0.5 |
